# EMSAR: estimation of transcript abundance from RNA-seq data by mappability-based segmentation and reclustering

**DOI:** 10.1186/s12859-015-0704-z

**Published:** 2015-09-03

**Authors:** Soohyun Lee, Chae Hwa Seo, Burak Han Alver, Sanghyuk Lee, Peter J. Park

**Affiliations:** 1 Department of Biomedical Informatics, Harvard Medical School, Boston, MA USA; 2 Emerging Technology Center, DNA link, Seoul, South Korea; 3 Ewha Womans University, Seoul, Korea; 4 Informatics Program, Boston Children’s Hospital and Division of Genetics, Brigham and Women’s Hospital, Boston, MA USA

**Keywords:** Expression quantification, Isoforms, Multi-reads, Optimization, Suffix array

## Abstract

**Background:**

RNA-seq has been widely used for genome-wide expression profiling. RNA-seq data typically consists of tens of millions of short sequenced reads from different transcripts. However, due to sequence similarity among genes and among isoforms, the source of a given read is often ambiguous. Existing approaches for estimating expression levels from RNA-seq reads tend to compromise between accuracy and computational cost.

**Results:**

We introduce a new approach for quantifying transcript abundance from RNA-seq data. EMSAR (Estimation by Mappability-based Segmentation And Reclustering) groups reads according to the set of transcripts to which they are mapped and finds maximum likelihood estimates using a joint Poisson model for each optimal set of segments of transcripts. The method uses nearly all mapped reads, including those mapped to multiple genes. With an efficient transcriptome indexing based on modified suffix arrays, EMSAR minimizes the use of CPU time and memory while achieving accuracy comparable to the best existing methods.

**Conclusions:**

EMSAR is a method for quantifying transcripts from RNA-seq data with high accuracy and low computational cost. EMSAR is available at https://github.com/parklab/emsar

**Electronic supplementary material:**

The online version of this article (doi:10.1186/s12859-015-0704-z) contains supplementary material, which is available to authorized users.

## Background

RNA-seq is a high-throughput sequencing-based technique for quantifying gene expression levels and for identifying splice isoforms, novel transcripts, sequence variation, and even fusion transcripts genome-wide. However, methods for expression quantification using RNA-seq are still not optimal—an important remaining challenge is to achieve maximal accuracy without a heavy computational load. One of the main difficulties in accurate quantification is the large amount of uncertainty inherent in the short-read data, where each sequenced read corresponds to a portion rather than the full length of an mRNA. This often causes ambiguity in the source of a sequenced RNA fragment, because a read can map to multiple locations in the genome or to a unique location that belongs to multiple isoforms. Production of multiple mRNA isoforms from a single locus is common in higher organisms: 92–94 % of human genes undergo alternative splicing [1]. Moreover, about 16 % of human genes have either close paralogs or pseudogenes [2], adding to the ambiguity in alignment. This problem is even more severe in other species, e.g., 70 % of soybean genes have a paralog due to extensive genome duplication [3]. Errors introduced during amplification and sequencing further increase the ambiguity. Therefore, to take full advantage of RNA-seq data, it is important to have a computational method that deals effectively with these problems. Current methods, however, involve a trade-off between accuracy and computational efficiency.

The expected count of the reads originating from a transcript is often modeled to be proportional to the abundance and length of the transcript. Though some approaches consider various additional factors for non-linearity such as biased amplification of cDNA fragments, the core component of quantification is this assumption of linearity. Some early approaches were not model-based. In Mortazavi et al. [4], the authors collected reads that mapped to each genomic locus, and normalized the count by the ‘gene length’, measured as the sum of exonic base pairs. Reads mapped to multiple loci (‘multi-reads’) were distributed among the loci according to the proportions of uniquely mapped reads at those loci. The resulting gene expression estimates were in the unit of RPKM (Reads Per Kilobase per Million reads). Although this work set the ground for RNA-seq-based expression quantification, its expression estimates were of moderate accuracy. Since a gene is often a composite of multiple isoforms with different expression levels and different lengths, using a single definition of ‘gene length’ inevitably lowers the accuracy. Moreover, it is difficult to extend this approach to isoform quantification.

Another early direct gene-level estimation approach, which we (S.L., C.H.S. and S.L.) developed in NEUMA [5], used only those reads mapping uniquely to the genic region that is common to all of its isoforms, and divided the count by the length of the common region. The approach showed relatively good performance for a transcriptome model when sufficiently large regions are common across all isoforms of a gene. For a complex transcriptome model, however, the estimation depends on a small fraction of reads, resulting in reduced accuracy and coverage. NEUMA quantifies isoform levels by dividing the number of reads unique to each isoform by the length of the unique region, and uses the sum of these isoform levels as an alternative gene-level estimate when possible. The major problem with this approach is that not all isoforms have sufficiently large unique regions.

Cufflinks [6] is one of the most widely used methods and also one of the earliest methods to resolve read ambiguity using multinomial distributions and maximum likelihood estimation. It estimates gene level abundance and isoform fractions within a gene independently. Some later methods align reads to the set of all transcript sequences (‘transcriptome reference’) rather than the genome, which allows more flexibility in quantification of transcripts by not having to group them by genomic loci. These methods, including eXpress [7] and RSEM [8], obtain a gene expression level as the sum of estimated isoform expression levels. However, in this strategy, the set of transcripts that share sequences must be quantified simultaneously, resulting in a large computational burden such as in RSEM, unless the algorithm is optimized.

The approach of modeling reads as multinomial-distributed variables and finding the fraction parameters for transcripts sharing reads using maximum likelihood estimation (MLE) is widely used for RNA-seq-based quantification. This is usually combined with expectation-maximization (EM) optimization, because the model involves a hidden variable that resolves the ambiguity of reads. The EM algorithm iterates between probabilistically assigning an ambiguous read to possible source positions and finding the abundance parameters. After it converges, the abundance parameters that best explain the overall read mapping are obtained. Cufflinks [6], eXpress [7], RSEM [8] IsoEM [9] and Seqem [10] are among the methods based on this Multinomial-MLE-EM scheme. Differences in the details of various approaches represent specific aims and trade-offs. Cufflinks achieves moderately fast and memory-efficient computation by not fully utilizing multi-reads and thus sacrificing some accuracy. The maximum likelihood estimation for isoforms is performed independently for individual genes to reduce computational cost. By default, this procedure uses unique reads only; optionally, multi-reads may be distributed in proportion to the estimated expression based on unique reads to improve accuracy. RSEM aims at achieving maximal accuracy by incorporating multi-reads at the cost of intensive use of computational resources. Both Cufflinks and RSEM use the batch EM algorithm that collects reads before performing the estimation and assignment, whereas eXpress uses the Online EM algorithm that updates the estimates as individual reads stream in to save memory and hard disk space [7]. However, the accuracy of eXpress is lower than Cufflinks and RSEM according to our comparison. IsoEM aims to optimize for speed by grouping reads that are shared by the same set of transcripts and then processing all the reads in that group simultaneously [9] However, we observed that IsoEM is memory-intensive and less accurate than the other methods.

Our new approach EMSAR (Estimation by Mappability-based Segmentation And Reclustering) adopts the transcript-first-gene-later approach based on transcriptome-mapped reads and uses nearly all of the reads including multi-reads (see Additional file 1 for details on exceptions). Our method models reads with Poisson distributions on segments of transcripts that share the reads, and identifies optimal sets of segments from the transcriptome to prebuild an index for fast and light processing of data. Although the underlying Poisson-based model is equivalent to the multinomial model (see below), partitioning of transcripts and parameter estimation from a joint Poisson model with no hidden variables is conceptually different, and therefore provides a unique opportunity for optimization that is not possible for the multinomial-based model. Since EMSAR’s Poisson model does not involve hidden variables, parameters can be estimated without the EM algorithm. We use a hill-climbing algorithm that allows user-specified precision (see Additional file 1). EMSAR uses multi-reads as RSEM does, but with a computationally efficient implementation. Although EMSAR’s strategy of grouping reads by the set of transcripts that they share is similar to the one adopted by IsoEM, one of the key differences is that EMSAR partitions transcript regions into segments so that each segment can be modeled using a single Poisson distribution with the expected read count proportional to the sum of expression levels of the shared transcripts times a pre-computed segment length. IsoEM does not partition transcripts and is based on the multinomial EM scheme.

Recent advances in expression quantification have focused on light-weight algorithms. For instance, eXpress [7] used an online EM algorithm to achieve fast computation with a small amount of memory. Sailfish [11] works on sequences directly without the need for alignment, by constructing a hash index of k-mers of transcriptome, at the expense of a large amount of memory (see below). However, elimination of the alignment step is likely to result in loss of information, since alignment helps in filtering spurious reads and provides information about fragment length distribution for paired-end data. EMSAR’s indexing preserves alignment-related information using modified suffix arrays [12] and a custom-designed, linked-list-based data structure. This indexing is light enough to be run on a personal computer—it takes only a few hours to process the human ENSEMBL annotations (one of the most complex trancriptomes) using 4 CPUs (see below) and less than 4GB of memory. Given an index and an alignment, EMSAR runs faster than Sailfish for individual RNA-seq data sets, as we will describe.

## Results and discussion

### Model and implementation

**Fig. 1:**
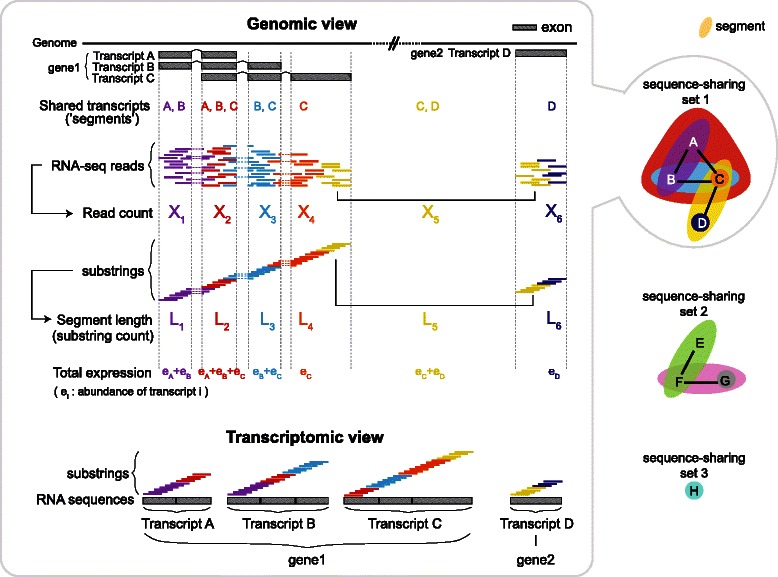
Principles and overall flow of EMSAR. An illustration of the key elements of EMSAR for single-end RNA-seq. Gene 1 has three splice isoforms and gene 2 has one. The two genes share some sequences, indicated by the yellow reads that are mapped to two locations. The RNA-seq reads are colored according to which transcripts share the read sequence. The read count (X_1_, …, X_6_) is the number of RNA-seq reads in the same ‘segments’ or that are shared by the same combination of transcripts. The length of the segments (L_1_, …, L_6_) correspond to the number of possible distinct virtual reads in each group. The read counts depend on the total expression level of the isoforms associated with each segment times the length of the segment. Transcripts are grouped into a sequence-sharing such that transcripts in the same segment belong to a set. A sequence-sharing set may contain transcripts from one or more genes. In this illustration, the top set (set 1) represents the transcripts shown on the left side. The four expression quantities (e_A_, …, e_D_) are estimated by considering the six segments simultaneously within this set

As illustrated in Fig. 1a, EMSAR groups reads according to the set of transcripts to which they map. Each such group of reads forms a “segment.” For example, a segment can be a set of reads that are mapped to one position on each of three transcripts A, B and C. Another segment can be a set of reads that are mapped to one position on B, C and to two positions on A, which may happen if there is an internal repeat on A.

Borrowing the concept of ‘virtual length’ introduced in early work by Sultan et al. [13] and used by NEUMA [5], EMSAR first defines a ‘virtual read’ as a subsequence of a transcript with the size of the read length (for paired-end data, it is a pair of such subsequences separated by a certain distance). Then, it counts the number of all possible distinct virtual reads in a segment and uses it as the (virtual) ‘segment length.’ When the fragment size distribution is available, such as with paired-end data or variable read-length single-end data, first the virtual length is computed for each fragment size, and then the overall segment length is computed as the average virtual length weighted by the fragment size distribution. The virtual length definition can easily be applied to any specific subset of reads, even when the regions covered by the reads are not continuous. Similarly, a ‘segment’ is virtual in that it is not necessarily a block of continuous nucleotides. The use of virtual length automatically adjusts for mappability and read length.

The read count for each segment is modeled as a Poisson variable whose expected value is proportional to the sum of the expression levels of all the transcripts defining each segment times the segment length. Then, for each set of transcripts that are either directly or indirectly linked by a segment (‘sequence-sharing set’), a likelihood function is defined as the joint Poisson function of all segments associated with the set. The abundance parameters of the transcripts are estimated simultaneously by maximizing this likelihood function. The sequence-sharing set is equivalent to the ‘bundle graph’ described in Roberts et al. [7]. When the set is larger than a certain size (e.g., 5000 transcripts), EMSAR removes segments with the length smaller than a threshold and rebuilds the sequence-sharing set. This process is repeated with an increasing segment threshold, until the maximum set size is smaller than the specified limit. It also restricts the number of alignments per read to 100 by default, since a read with such a large number of alignments contains little information.

EMSAR uses a hill-climbing algorithm for the maximization. All the segments in the transcriptome and their fragment-size-specific lengths are pre-computed as an index to minimizes computational time. The index is built efficiently by using a modified suffix array and other data structures (see Methods and Additional file 1 for details).

The algorithm produces output in units of FPKM (Fragments per Kilobase per Million reads) and TPM (transcripts per million). These units were introduced by Cufflinks [6] and RSEM [8], and are more accurate than the earlier RPKM in that they consider the gene level expression as the sum of isoform level expression. EMSAR also uses FPKM and TPM values to infer and reports read counts for individual transcripts and genes.

### Handling multi-reads

One of the technical decisions to be made when designing an RNA-seq-based quantification method is what to do with reads that are shared by multiple genes (multi-reads). Considering the significant proportion of paralogs and pseudogenes in most genomes, making good use of multi-reads is essential for highly accurate estimation. However, using multi-reads involves simultaneous quantification of a large number of transcripts or genes, which results in increased computational cost. EMSAR utilizes multi-reads as efficiently as reads unique to individual genes; yet, due to the unique segmentation-based design, it also achieves a light-weight implementation through optimized data structure.

**Fig. 2:**
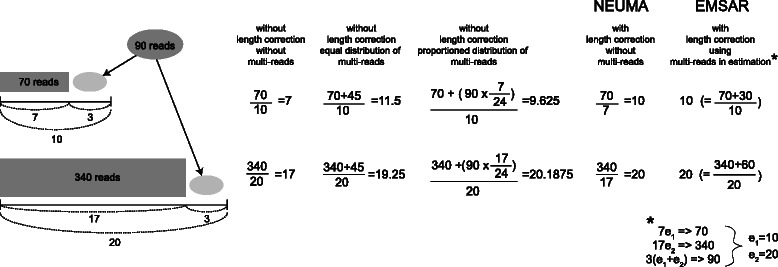
An illustration of the effect of various length correction approaches. On the left is shown a schematic of two genes that share some sequences, along with the lengths of the unique and shared regions and the numbers of reads mapped to these regions. The right panel shows the estimated abundance using five different length correction strategies

In Fig. 2, we illustrate five different approaches for handling multi-reads with a simple case of two genes having single isoforms of length 10 and 20, respectively (unspecified unit), and sharing a multiply-alignable region of length 3. A total of 70 and 340 reads are uniquely mapped to each gene, respectively, and 90 reads are mapped to both. Intuitively, the 90 multi-reads should be divided between the two genes in accordance with the read density observed in the uniquely mappable regions. That is, there are 10 reads per unit for the short gene (top) and 20 reads per unit for the long gene (bottom), and so the 90 reads should be divided with 1:2 ratio, with 30 reads to the short gene and 60 reads to the long gene. These expression levels are formulated as the maximum likelihood solution to three Poisson likelihood equations involving two unknown parameters shown in Fig. 2 (lower right corner).

An approach without an appropriate length correction and ignoring multi-reads would produce abundance estimates of 7 and 17 by normalizing by the full length of the genes. Cufflinks distributes these 90 reads equally (45 vs 45) by default, or in proportion to the estimates computed using the total gene length with the -u option [7]. NEUMA, with length correction using unique reads only, would divide the read count by 7 and 17 instead of 10 and 20, respectively, producing an unbiased result of 10 and 20. EMSAR employs a proper length correction while utilizing multi-reads. The differences between the methods were not substantial in Fig. 2, but for genes with low mappability (due to belonging to large gene families or having paralogs), the impact of ignoring or inadequately correcting for multiply-aligned regions is larger.

### Comparison of accuracy in isoform level quantification using simulation

It is important to assess the performance of different algorithms in estimating transcript abundance at the isoform level. We generated RNA-seq reads using FLUX Simulator [14], which creates a simulated transcriptome by randomly assigning expression levels to transcripts from a theoretical distribution of expression levels and simulates experimental steps including RNA fragmentation, PCR, size selection and sequencing. Figure S1 shows the distribution of the read positions and fragment lengths in one of our simulated datasets. We compared EMSAR to Cufflinks, eXpress, RSEM, IsoEM, Sailfish and NEUMA using 5 to 40 million unstranded single-end and paired-end reads of length 101 bp. These reads were generated from a randomized human transcriptome based on the ENSEMBL GRCh37.72 annotation with a total of 194,701 RNA species associated with 57,231 genes.

The assessment was performed with the true expression levels generated by the simulator as the gold standard, using several measures including the Pearson correlation coefficient, root mean square error (RMSE, not to be confused with the method RSEM), number of false positives and false negatives. Seqem was not compatible with our test because of the limit on the number of genes imposed by the software. To remove the undue influence of large values, we compared the numbers in the log scale: log(σM_e_ + 1) versus log(M_t_ +1), where M_e_ is the estimated TPM ($FPK{M}_i/{\displaystyle {\sum}_{i\in T}}FPK{M}_i\times {10}^6$ for transcript *i* in the transcriptome *T*) and M_t_ is the molecular fraction of the transcript × 10^6^. A scaling factor σ is introduced in each method as an adjustable parameter that maximizes correlation coefficient among non-zero expressed and non-zero estimated data points. For RMSE, linear regression was used to minimize the sum of squared errors for each method (see Method for more details). Since some programs do not estimate all transcripts, we also had to choose which set of transcripts to use for comparison. For this, we adopted two different strategies. First, we used the set of transcripts that are estimated by all of the programs (‘commonly estimated set’). Second, we performed pairwise comparisons using all of the transcripts estimated by both EMSAR and the method being compared. Since EMSAR reports estimates for all transcripts, this is simply the set of transcripts estimated by the other method.

**Fig. 3:**
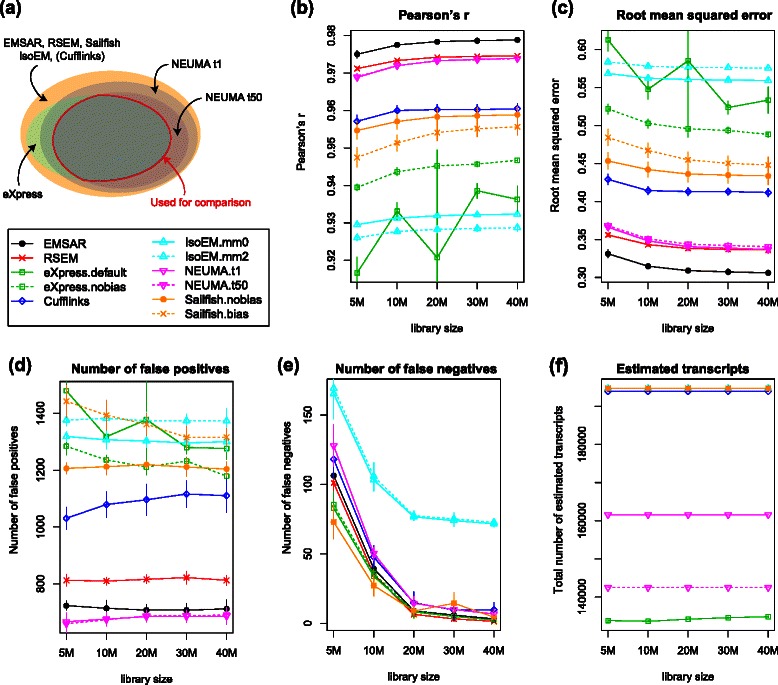
Comparison of accuracy across multiple methods for single-end RNA-seq. **a** A schematic showing the set of transcripts estimated by individual methods and the common set (not drawn to scale). **b**-**e** Four different evaluation criteria are applied on the common set of transcripts indicated in (**a**). IsoEM.mm0 and IsoEM.mm2 refer to IsoEM runs with up to 0 and 2 mismatches allowed, respectively. NEUMA.t1 and NEUMA.t50 refer to NEUMA runs with EUMA (length) cut-off 1 bp and 50 bp, respectively. eXpress.default and eXpress.nobias refer to eXpress runs with default and –no-bias option, respectively. (**b**) Pearson correlation coefficient (**c**) root mean squared error (**d**) number of false positives, i.e., isoforms with zero true expression and with 1 or larger value in log_2_(σM_e_ + 1), where M_e_ is the estimated TPM ($FPK{M}_i/{\displaystyle {\sum}_{i\in T}}FPK{M}_i\times {10}^6$ for transcript i in the transcriptome T), (**e**) number of false negatives, i.e., isoforms with 1 or larger value in log_2_(σM_t_ + 1), where M_t_ is the molecular fraction of the transcript × 10^6^, and zero estimated expression. (**f**) total number of transcripts whose expression level is reported. EMSAR, RSEM, Sailfish and IsoEM report all transcript levels. Error bars indicate standard deviation, not standard error of mean

**Fig. 4:**
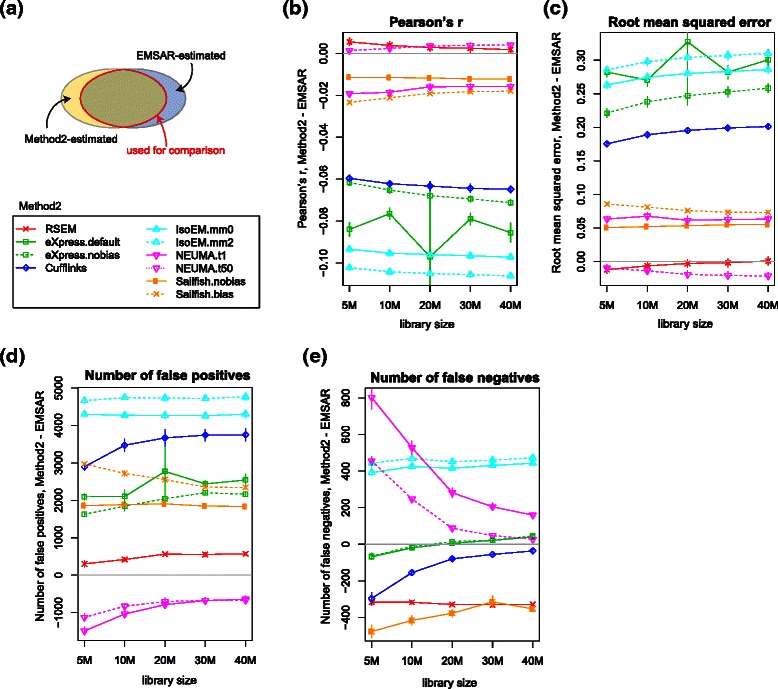
Pairwise comparison of accuracy between EMSAR and other methods for single-end RNA-seq. **a** A schematic showing the set of isoforms common to EMSAR and the other method (not drawn to scale). **b**-**e** Similar to Fig. 3, except that the differences between the other method and EMSAR are plotted

Figures 3 and 4 show the results for single-end data. Based on the commonly estimated set of transcripts, EMSAR showed the best performance when measured with the Pearson correlation coefficient and RMSE, followed by RSEM, Cufflinks, Sailfish, eXpress and IsoEM (Fig. 3). This result is as expected, since RSEM and EMSAR treat multi-reads more comprehensively than Cufflinks and eXpress, and Sailfish and eXpress sacrifice accuracy for lighter computation. IsoEM did not perform as well in our analysis as in the previous comparison by Li et al. [7]. EMSAR produced fewer false positives but slightly more false negatives than RSEM, though the differences in false negatives were small across all programs except IsoEM. Sailifish shows a low false negative rate. It should be noted that this transcript set is mostly constrained by NEUMA and eXpress since abundance estimates were missing for about 30,000 ~ 50,000 transcripts with NEUMA and about 60,000 transcripts with eXpress (Fig. 3f). NEUMA quantifies only transcripts with at least some uniquely mappable sequence, and eXpress flags transcripts as solvable when reads are assigned to them. Therefore, this common set of transcripts consists of relatively easy cases.

For pairwise comparisons shown in Fig. 4, we plotted the difference (rather than the absolute values) in each metric between EMSAR and the other program, since a different set of transcripts is used in each case. The performance of EMSAR was comparable to that of RSEM in this setting with respect to the Pearson correlation and mean square error. Again, EMSAR produced fewer false positives and more false negatives compared to RSEM. Since both RSEM and EMSAR report abundance for all transcripts, this particular comparison covers the entire transcriptome. Consistent with the result from the commonly estimated set, Cufflinks, eXpress and IsoEM showed increasingly larger differences compared to EMSAR and RSEM. Sailfish did better than Cufflinks in this comparison, but it was slightly outperformed by EMSAR.

Comparisons on paired-end data showed similar results (Figures S2 and S3). One difference is that Cufflinks somehow performed very poorly and showed an extremely skewed relation to true expression level, as indicated by a high σ value (see below) and a low correlation coefficient of r = 0.7 ~ 0.75. Figure S4 shows scatter plots between true and estimated expression levels for both single-end and paired-end. The scatter plots showed a secondary cluster of transcripts with a different relationship to the true expression level. This may be due to an implementation issue. Li et al. [8] also pointed out that Cufflinks had surprisingly poor accuracy on the Ensembl set with abnormally high abundance estimates for a subset of short transcripts. Sailfish did similar to or worse than eXpress and produced the largest number of false positives. This may be because the k-mer counting approach in Sailfish is suitable for single-end data but it does not take advantage of the additional information provided by paired-end libraries.

The consistently higher accuracy obtained by EMSAR and RSEM is likely due to their efficient treatment of multi-reads. Some differences between these two methods include bias modeling in RSEM and a special alignment adjustment strategy (described below), more precisely calculated lengths of source transcript region, and data-specific fragment size distribution in EMSAR.

EMSAR currently does not incorporate bias due to GC% or relative position on the transcript into the model, as RNA-seq techniques have been improving over time to compensate for positional bias, with methods such as dUTP [15, 16] offering a fairly uniform coverage [17]. Interestingly, we note that modeling this bias does not necessarily improve the result. For instance, the overall accuracy of eXpress was better with the “*–no-bias*” option for the simulated data even though these data contained some positional bias (Figure S1). This suggests that having a bias model does not always help and may even harm the performance if the model does not fit the data well. How to best model various biases of RNA-seq data remains an interesting question.

### Capturing systematic bias with the sigma parameter

The purpose of the method-specific scaling was mainly to avoid penalizing methods that do not report estimates for all transcripts or methods that may produce a systematic bias. Deviation of σ from 1 may indicate that the method’s reported sum of all transcript expression is underestimated due to incomplete coverage, which would boost the TPM value, or that the method’s estimation procedure generated some systematic bias resulting in a non-linear relationship between the estimated and true expression levels. Cufflinks showed the largest deviation from σ = 1, though it is not clear what caused this deviation. NEUMA and eXpress with the “--no-bias” option tend to have σ lower than 1, consistent with their underestimated ΣFPKM. However, eXpress with default options (with a bias model) occasionally had high sigma values, indicating systematic skewness in the estimated expression levels. EMSAR and RSEM consistently had σ close to 1, which suggests that introducing the σ factor provided the least benefit for EMSAR and RSEM in our comparisons described above. The values for σ and the maximized correlation coefficient among non-zero transcripts are shown in Figure S5.

### Comparison of speed and memory usage

**Fig. 5:**
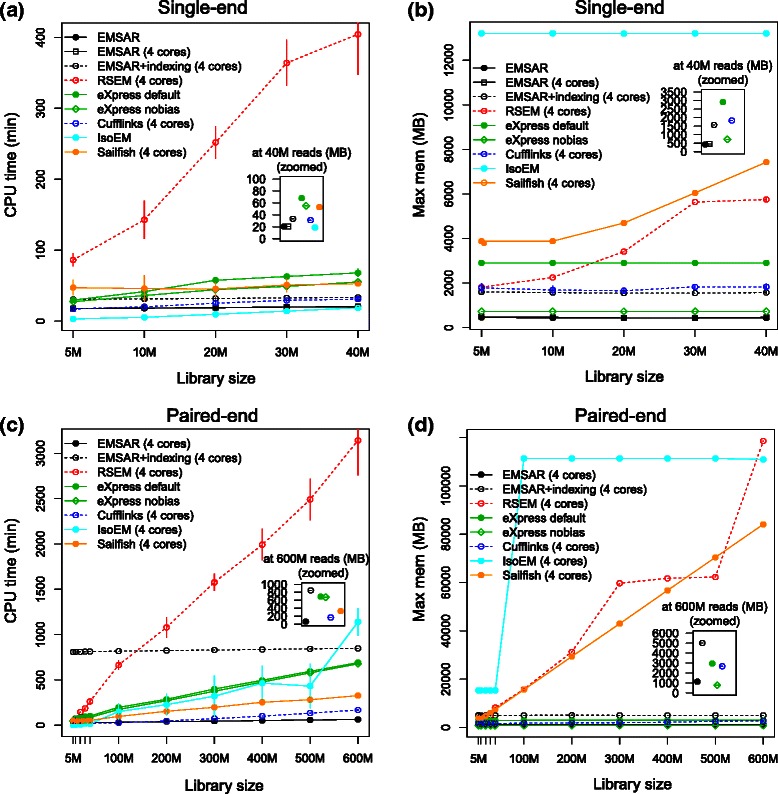
Comparison of running time and memory usage with respect to library size. **a** CPU time and (**b**) maximum memory usage for single-end data, (**c**) CPU time and (**d**) maximum memory usage for paired-end data, measured on a machine with Intel Xeon 2.90 GHz CPUs. EMSAR, RSEM and Cufflinks were run with 4 slots for shorter run time; EMSAR was also run without multi-threading for the single-end case. IsoEM did not provide a parallelization option. Alignment process is not included in the comparison

Speed and memory usage are important considerations in choosing an algorithm, especially if the sequenced library size is large. For single-end data (Fig. 5a, b), EMSAR’s speed and memory usage was among the best; for instance, EMSAR was slightly faster than eXpress and Sailfish and similar to IsoEM. Given an index, EMSAR’s memory usage was the lowest; when index creation is included, EMSAR was the second lowest, only behind eXpress (run with the “no-bias” option). IsoEM was the fastest but used over 12GB of memory. These results for IsoEM were obtained after we modified IsoEM’s java memory option to use 8GB only. Without this modification, the program attempted to use more memory than available and produced erroneous results. RSEM, which was comparable to EMSAR in terms of accuracy, required the most CPU time. In particular, its CPU time increased steeply with respect to the library size, and was more than 10 times greater than that of EMSAR for libraries with 30–40 million reads. The results also show that EMSAR gets nearly maximal benefit from multi-threading. The total CPU time does not increase when 4 cores are used instead of 1, suggesting that the multi-threading overhead of EMSAR is negligible. This is also true for paired-end data (not shown).

For paired-end data (Fig. 5c, d), EMSAR’s indexing uses more CPU time and therefore there is some disadvantage for a small library, but the efficiency increases with library size. For RSEM, both CPU time and memory usage increased steeply with library size, as with the single-read data, and a modification of memory requirement was needed to run on very large data sets. Sailfish was similarly memory-intensive, particularly for large libraries, although it was faster than eXpress. EMSAR data processing was the fastest of all the programs once the indexing had been done. Our results on Cufflinks, eXpress and RSEM are consistent with what has been reported previously, except that we observed a faster performance of Cufflinks [7]. The Cufflinks runs with small CPU time associated with spuriously low accuracy (see above) were repeatedly observed on our simulation data, but not in a different data set (see below *Multi-sample performance*). IsoEM needed a memory request of larger than 10GB for libraries larger than 100 million reads. We requested to use maximum 100GB for those libraries, and all of the requested memory was used. In summary, our comparison study indicates that EMSAR is superior to RSEM in terms of efficiency in computational resources while having similar accuracy.

### Multi-sample performance

**Fig. 6:**
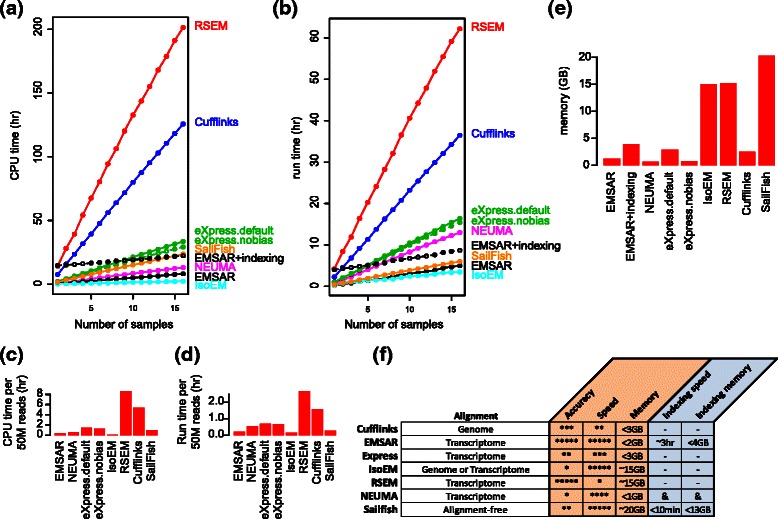
Comparison of running time and memory usage with respect to number of samples. **a** CPU time, (**b**) run time and (**c**) maximum memory usage on a serial run of multiple human paired-end RNA-seq data of library size 60–80 million reads, measured on a machine with Intel Xeon 2.90 GHz CPUs. The X values of (**a**) and (**b**) are incremented as each library (in an arbitrary order) is added to the run. The slopes of (**a**) and (**b**) are plotted in (**d**) and (**e**), respectively, in hours per 50 million reads. Alignment process is not included in the comparison. (**f**) A chart that summarizes quantification programs based on our results, in relative accuracy, speed and memory for data processing and speed and memory for indexing

We also evaluated the performance of the above programs on 16 human paired-end RNA-seq data available from a public repository (http://www.ncbi.nlm.nih.gov/geo/query/acc.cgi?acc=GSE55504, [18]). Each of the 16 samples contains 60–80 million reads. Figure 6 shows CPU time (Fig. 6a), run time (Fig. 6b), and maximum memory usage (Fig. 6c), when the 16 samples were processed serially using 4 cores (with the exception of eXpress which was run using 1 core). Figure 6d and e summarize the slopes of Fig. 6a and b, respectively, in hours per 50 million reads. Consistent with the comparisons on simulated RNA-seq data, EMSAR shows an outstanding speed. In particular, the CPU time of EMSAR is similar to that of Sailfish for the 16 samples, indexing time included. With prior alignment and indexing, the data processing was faster with EMSAR than Sailfish. It took more than 2 days for RSEM to finish the runs while EMSAR needed less than 4 hours given an index. Cufflinks took longer (1.5 days), in contrast to our observations with simulated data. In terms of memory, EMSAR was one of the lightest performers (less than 2GB memory for data processing, less than 4GB for indexing), whereas Sailfish, RSEM and IsoEM used a large amount of memory (15–20GB). Overall, EMSAR is light enough to be run on a personal computer and the advantages of EMSAR are particularly pronounced when working with multiple samples as well as large libraries. A summary comparison chart based on our results is provided in Fig. 6f, and it highlights high accuracy and computational efficiency of EMSAR.

### Comparison of accuracy in gene level quantification to qPCR

We also examined the performance of EMSAR in quantifying gene expression level from real RNA-seq data from samples with qPCR data available. For our main comparison, we ran EMSAR, Cufflinks, eXpress, RSEM, IsoEM, Sailfish and NEUMA on four RNA-seq datasets of UHRR (universal human reference RNA) with two transcriptome models (RefSeq and ENSEMBL (GRCh37.73)), and compared the results to qRT-PCR (TaqMan) data by two different groups (MAQC (Microarray quality control), 1001 genes [19]; Wang et al., 1363 genes [20]). This resulted in 6 algorithms x 4 samples x 2 models x 2 qRT-PCR sets = 96 data points. Furthermore, we performed the same analysis for two additional samples: HBRR (human brain reference RNA) with TaqMan qRT-PCR data, and a gastric cancer cell line MKN-28 with SYBR qRT-PCR data [5]. The details of the RNA-seq and qRT-PCR data used are provided in Additional file 1: Tables S2 and S3. In each case, we computed the Pearson correlation coefficient between RNA-seq estimates with qRT-PCR measurements (details in Materials and Methods).

**Fig. 7:**
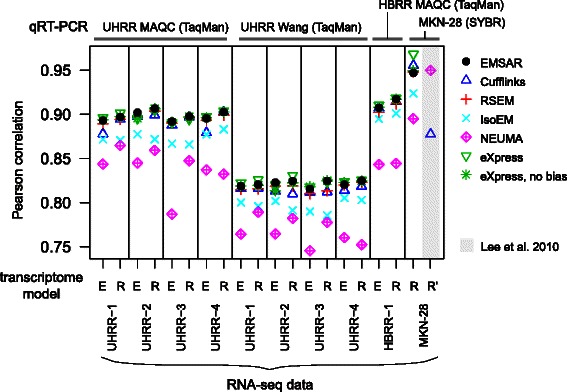
Comparison of accuracy as measured by concordance to qRT-PCR on real RNA-seq data. Pearson correlation between RNA-seq-based gene expression level estimates (log(TPM*τ + 1)) and qRT-PCR-based measurements (ΔCt), across independently performed experiments. τ was used to maximize the correlation to adjust for any scale effect of the pseudocount, which allowed inclusion of zero estimates. Six RNA-seq data sets (UHRR-1 to −4, HBRR-1, MKN-28) and four qRT-PCR sets (UHRR MAQC [19], HBRR MAQC [19], UHRR Wang et al. [20], MKN-28 from Lee et al. [5]) were compared. RNA-seq-based quantification was run on two independent transcriptome models (ENSEMBL (E) and RefSeq (R)). The results from Lee et al. on an older version of RefSeq model (R’) was shown to the right for comparison (grey box). Gene-level estimates were obtained by summing the relevant isoform-level estimates, except for NEUMA, for which gene-level estimates were obtained either from the reads common to all the isoforms of a gene or by summing the isoform-level estimates derived from reads unique to individual isoforms

The result shown in Fig. 7 indicates that EMSAR, eXpress, and RSEM are the top-performing methods overall, with Cufflinks slightly behind. IsoEM, Sailfish and NEUMA consistently show lower concordance between qRT-PCR and RNA-seq. Sailfish produced poor results for UHRR1 and UHRR2, with r < 0.45 (shown in Figure S6). Interestingly, the major determinant of the concordance level is the choice of qRT-PCR dataset (~ 0.83 vs ~ 0.90 for the two qRT-PCR sets), rather than the quantification method, the transcriptome model, or even the type or library size of the RNA-seq datasets. Both MAQC and Wang et al. qRT-PCR datasets are based on TaqMan, which defines a primer pair and a junction probe as a single assay and applies multiple assays to quantify a single multi-exon gene. On the other hand, the qRT-PCR dataset in Wang et al. was based on a single assay for each gene. This may explain the low concordance rate between the RNA-seq and qRT-PCR measurements from Wang et al. For HBRR, the results are similar to those on UHRR from MAQC, as they were based on the same qRT-PCR assay. The qRT-PCR for MKN-28 was based on 27 genes, randomly selected from each of eight expression quantile groups among the genes whose expression was confidently reported by four methods, NEUMA, Cufflinks, Tophat and ERANGE [5], and the primers were designed to cover the exon junction that is common to all isoforms for each gene (as opposed to integrating values from multiple exon junctions including non-common ones). The concordance rate for this data set and the RNA-seq data was higher than the other datasets, presumably because the gene set is well-balanced across a wide expression range and the gene-level qRT-PCR measurement was straightforward.

The comparison between ENSEMBL- and Refseq-based runs shows that Refseq-based runs exhibit better concordance with the qRT-PCR overall. This may be explained by the fact that all of the qRT-PCR primers used here were designed based on RefSeq annotations. The substantial decrease in NEUMA’s performance with the ENSEMBL model could partly be explained by the inherent limitation of the method, which relies on the regions common across all isoforms of a gene. Its performance would suffer significantly as the transcriptome model becomes more complex. Similarly, the discrepancy between its performance on the new run of MKN-28 and the performance reported in the 2010 paper may be explained by the fact that the Refseq transcriptome has become more complex now. The 2010 version had 18,909 protein-coding genes with 29,754 isoforms, whereas the current version has 25,497 genes with 47,308 isoforms. On the other hand, Cufflink’s performance shows a drastic improvement on the same MKN-28 data set over the 2010 comparison. This may be partly due to improvement in implementation, incorporation of bias models and incorporation of multi-reads (note that we ran cufflinks with *–u* option which uses multi-reads to improve accuracy).

A recent SEQC (RNA sequencing quality control) project led by the MAQC consortium compared RNA-seq, microarray and qPCR. The concordance between two different qPCR experiments on 843 genes was quite low (correlation coefficient 0.85 ~ 0.86), implying that using qPCR as a quantitative gold standard has significant limitations [21].

### Comparison of alignment strategies when allowing mismatches

It is a common practice to allow 1 ~ 2 mismatches when mapping RNA-seq reads to a reference genome or transcriptome, to account for sequence variations in the individual sample as well as sequencing error and to increase the number of mappable reads. It is difficult to compute precise segment lengths in the presence of mismatches, i.e., enumerating all possible reads with and without mismatches with proper weighting by an error model. However, we reasoned that if allowing mismatches does not alter the proportion of reads mapped to distinct segments, we can use the segment length computed with the assumption of perfect mapping, for mismatch-allowed read counts.

**Fig. 8:**
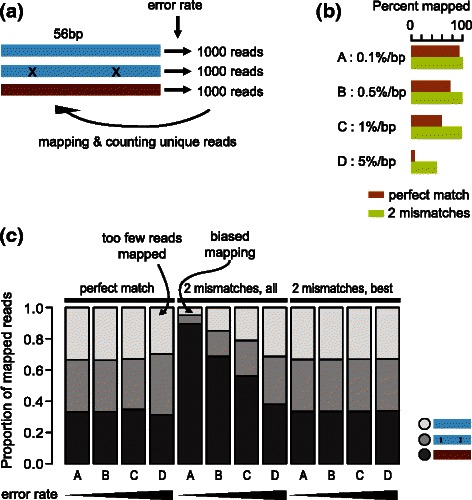
Simulation of mapping with mismatches. **a** In this simulation, three 56-bp artificial gene sequences were created. Two sequences (blue) were identical except at two nucleotide positions (marked as X, position not in scale). From each artificial gene, 1000 full-length reads were generated with base substitution errors. The reads were mapped back to the reference, and only uniquely mapped reads were accepted. **b** Four different error rates used for the simulation and the resulting mapping percentage, either when only perfect match is allowed (brown) or when up to two mismatches are allowed (green). **c** The fractions of uniquely mapped reads in the three genes illustrated in (**a**). The grey scale indicates one of the three genes. The column labels A-D indicate independent simulations with the error rates indicated in (**b**). ‘Perfect match’ allowed only perfect matching alignment. ‘2 mismatches, all’ accepts all alignments up to 2 mismatches. ‘2 mismatches, best’ accepts all the alignments with the minimum number of mismatches for each read

We investigated this possibility by simulating 1000 artificial full-length RNA-seq reads from two nearly identical sequences and one very different one (a ‘transcriptome’ with three transcripts, each 56 bp in length), with various degrees of sequencing errors. We mapped them back to the reference allowing mismatches, and selected uniquely mapped reads (Fig. 8a). As expected, for the four different error rates we tried (0.1, 0.5, 1, and 5 %), the percent of mapped reads decreased as the error rate increased (Fig. 8b). In this simulation, there are three segments and each of them is unique to a transcript and has length 1 (length 1 because the transcript length is identical to the read length). If only a perfect match were allowed, reads would be aligned only uniquely to a single transcript. However, with mismatches, a read may map to multiple transcripts. We discarded such reads, since we do not have the corresponding multi-transcript segments. Since we generated the same number of reads from each transcript, alignment strategies can be evaluated by checking whether the three reads are equally distributed across the three transcripts.

We compared three alignment strategies: 1) retrieving all perfectly-matched alignments exclusively (‘perfect match’), 2) retrieving all alignments allowing up to 2 mismatches (‘2 mismatches, all’), and 3) retrieving all alignments with the fewest mismatches for each read (‘2 mismatches, best’). When we considered only perfect matches, the read counts for the three sequences were nearly identical, indicating no bias (Fig. 8c). However, with increasing error rates, the percentage of noise also increased because the number of mapped reads decreased. The ‘2 mismatches, all’ strategy produced a highly biased result, particularly when the error rate was low. This is because the mapping of reads originating from the two nearly identical genes became ambiguous when mismatches were permitted, reducing the number of uniquely mapped reads disproportionately. When the error rate is low, reads can be mapped to the true location even without allowing mismatches, and therefore allowing mismatches introduces additional ambiguity. When the error rate is high, allowing 2 mismatches helps find the true location and does not introduce as much ambiguity because alignments with fewer mismatches are less likely. With the ‘2 mismatches, best’ approach, the ambiguity introduced by allowing extra mismatches can be avoided, and the correct proportion of uniquely mapped reads can be restored. At a higher error rate, this method works better than the ‘perfect match’ strategy, because using more reads reduces noise.

These results suggest that, in general, allowing mismatches can distort uniquely mappable proportions among highly similar sequences, and may affect quantification based on read counts. Based on these observations, EMSAR internally filters alignments to retain only the best-matching ones, with the assumption that all of the alignments up to a certain mismatches are reported. In other words, it takes ‘*n* mismatches, all’ alignments as input and internally filters them into a set of ‘*n* mismatches, best’ to use for quantification.

### Relationship between statistical models

The underlying segment-wise Poisson model used by EMSAR is statistically equivalent to the joint Poisson model at individual base positions [22]. The maximum likelihood estimator of the joint multinomial model at the individual read level employed in Cufflinks is equivalent to that of the joint Poisson model, owing to the well-known equivalence of multinomial and conditional Poisson distribution [23]. Thus, the joint Poisson model used by EMSAR is, at its core, statistically equivalent to these other models.

We have used this underlying model to produce an efficient implementation with minimal sacrifice in accuracy. We also achieved other improvements by effective use of multi-reads, balanced alignment filtration, use of data-specific fragment-length distribution, and optimization by a hill-climbing algorithm with user-selected precision. Combining all these features has made EMSAR an accurate yet computationally efficient method.

### Implementation and availability

EMSAR is available as a C program and takes an alignment file (SAM, BAM and default Bowtie1 output format) and a transcriptome FASTA file as input. The alignment file can be streamed. The main output file contains FPKM and TPM values and inferred read counts for individual transcripts. A multi-threading option is implemented as well.

## Conclusions

We have developed a method that implements a novel optimization procedure for expression estimation from RNA-seq data. Our method avoids the EM-based probabilistic transcript assignment for individual reads and instead counts reads in each read group defined based on transcript sharing. Our method achieves high accuracy, comparable to that of RSEM and better than the rest of the methods we tested. Importantly, our method also achieves superior speed and memory usage, by using a pre-built transcriptome index. These results suggest that EMSAR is a favorable alternative to existing methods in many situations.

## Methods

### Model and algorithm

#### Terminology

**A transcript** is a sequence that corresponds to a full length RNA species. The transcriptome refers to the set of all transcripts defined for an organism. Note that here, by a transcript we mean a full-length RNA species with a distinct sequence, rather than a direct molecular output of transcription as traditionally used in biochemistry and molecular biology.

For single-end RNA-seq, **a read** is a sequence from one end of a transcript fragment in the data. For paired-end RNA-seq, a read is a pair of sequences from both ends of a transcript fragment in the data.

For paired-end RNA-seq, **a fragment size (or fragment length)** is computed as the end-to-end distance of the two mates of a read when aligned on a transcript. Multiple possible fragment sizes may exist for a given read. We use only reads with a unique fragment size (see Additional file 1 for details). For single-end RNA-seq with variable read lengths, the read length is treated as fragment size, since most of these cases are fragment size shorter than the number of bases sequenced and the variable read length is obtained by removing the 3’adaptor sequence. For single-end data with a fixed read length, again the read length is treated as the fragment size since it can be considered the minimum fragment size and the actual distribution of fragment size is unknown.

An **alignment class** is a set of transcripts and the number of positions on each transcript that a read is mapped to (e.g., [t_1_,t_2_,t_3_,t_3_] can define an alignment class where t_1_,t_2_ and t_3_ are distinct transcripts).

A **virtual read** is defined as a subsequence of a transcript, with the size of the read length (for single-end) or a pair of subsequences with the size of the read length, separated by a certain distance (for paired-end).

The set of all possible distinct virtual reads mapped to an alignment class is a **segment**. The **length of a segment** is the number of distinct virtual reads that form the segment. When multiple fragment sizes are present, each fragment-size-specific segment length is computed first and then the overall segment length is computed as the average segment length weighted by the probability of each fragment size.

The **read count of a segment** is defined as the total number of reads in an RNA-seq data in that segment. Identical reads may be counted multiple times if they occur multiple times in the data. The read count is modeled as a Poisson random variable whose expected value is proportional to the sum of expression levels of all the transcripts associated with the segment, (e.g., ${e}_{t_1}+{e}_{t_2}+{e}_{t_3}+{e}_{t_3}$ for the segment associated with alignment class [t_1_,t_2_,t_3_,t_3_], where *e*_*i*_ is the abundance of transcript *i*) times the segment length.

**A sequence-sharing set** is defined as the minimal set of transcripts that ever share the same segment, i.e., the same alignment class. In other words, a sequence-sharing set S is the smallest set S_0_ that satisfies the following condition for transcripts u and v in transcriptome T: if there exists a alignment class Z such that u∈Z and v∈Z, then u∈S_0_ and v∈S_0_. Sequence-sharing sets are mutually exclusive and their union is the entire transcriptome.

Likewise, an equivalence set G of segments that ever share a transcript can be defined as the smallest set G_0_ that satisfies the following condition: for segments u and v in the set of all segments Q, if there exists a transcript that both u and v are associated with, then u∈G_0_ and v∈G_0_.

#### Maximum likelihood estimation

We model the segment read counts as a Poisson random variable that depends on the sum of the expression levels of the transcripts sharing the segment, the segment length, and the total sequencing depth.

We define a likelihood function for read count X_c_ for segment C as *LH*(e; X_C_) = Poisson (λ), where $\lambda = \left({\displaystyle {\sum}_{i\in Z}}{e}_i\right){L}_CM,{e}_i$, is the abundance of transcript *i* in the alignment class Z associated with segment C, *L*_*C*_ is the segment length, and M is a scale factor proportional to the total number of reads in the experiment. Then, we maximize ${\displaystyle {\prod}_{C\subset G}}LH\left(\mathrm{e};\ {\mathrm{X}}_{\mathrm{C}}\right)$ over all segments encompassed by the equivalence set G. Note that we used set operations here for convenience, though an alignment class is not a set in that the same element can appear multiple times.

For maximization, we use an efficient hill-climbing algorithm. We report the expression levels for individual transcripts. The optimization is performed multiple times (by default, 4 times) with different random initial points and the mean is reported. This way, if a transcript is unsolvable but the sum of two transcripts is solvable, then the two transcripts will be estimated to be expressed at about the same level.

#### Inference of read counts

After computing the FPKM value, the read count for transcript $i$ is inferred as:

$$ \hat{Readcoun{t}_i}=\left({\displaystyle \sum_{\forall C}}{X}_C\right)\frac{FPK{M}_i\left({\displaystyle {\sum}_{i\in C}}{L}_C\right)}{10^9} $$

Where C refers to a segment, with segment read count *X*_*C*_ and segment length *L*_*C*_. The term ${\displaystyle {\sum}_{i\in C}}{L}_C$, or the sum of the lengths of all segments that include transcript *i*, is the effective transcript length for transcript *i*. Theoretically, this should be identical to the actual transcript length, since EMSAR uses all of the reads. However, for practical reasons, we exclude reads and segments that are shared by more than 100 locations. For this reason, there may be a slight difference between the effective length and the actual length. A gene level read count can be computed as the sum of isoform read counts. These read counts can be fed to differential expression analysis programs [24–27], or renormalized using any count-based normalization methods [28].

#### Algorithm

A modified suffix array is built on a concatenated transcriptome, so that identical sequences of a specified length are clustered on the array (Figure S7a). A suffix array is an array of all positions in an input string (here the concatenated transcriptome) sorted by the suffix starting at each position. We modified the suffix array so that the sorting considers only the first portion of each suffix up to the read length and so that reverse complementary sequences are not separated into distinct clusters for unstranded RNA-seq data. Identical substrings are clustered in our modified suffix array, and for each cluster, EMSAR converts the positions on the concatenated transcriptome to the corresponding transcript ID using a separate index table, then either creates a new segment element in a sorted linked list accessed by a 2-dimensional array or increment the length of an existing segment.

For paired-end RNA-seq, a similar modified suffix array is created for one of the two mates. Then, for each cluster on the first array, a secondary array is created temporarily, representing all possible positions of the other mate for a given range of fragment sizes. A cluster on the second suffix array represents positions of identical second mates on the transcriptome. Since it is conditioned on a cluster of identical first mates, it represents identical paired-end reads. The subsequent steps are the same as in the single-end case (Figure S7b).

The data structure used for the index is shown in Figure S8a. The index contains information on the length of each segments computed for individual fragment lengths The final segment length is completed for each RNA-seq data, using its data-specific fragment length distribution. Once all segments are identified, the sequence-sharing sets are computed using a recursive propagation algorithm (detail in Figure S8b). When the sequence-sharing set is larger than a threshold, segments with length below a threshold are iteratively dropped and the sets are recalculated.

### Parameters used in program runs

The FLUX simulator was run with the following settings to generate two independent random transcriptomes based on the human ENSEMBL annotation GRCh37.72. From each transcriptome, we added random differential expression to create two additional sets. Then, unstranded paired-end RNA-seq data of various sizes with read length 101 bp were generated from each transcriptome. For single-end data analysis, we used one of the two mate files. Additional parameters used for FLUX simulator can be found in Table S1.

EMSAR 2.0.0 was run with the default parameters, along with Bowtie 1.0.0 with options *-v 2 -a -m 100 -f -p 4 -S*. This setting allows two mismatches.

eXpress 1.3.1 was run with default options. An additional run with option *--no-bias-correct* was included. For mapping, Bowtie 1.0.0 was used with parameters *-aS --offrate 1 -X 800 -v 2 -f -p 4* as recommended on the program web site.

For Cufflinks, version 2.1.1 was used with default options, along with Tophat 2.0.8b with options *-p 4 -N 2 --bowtie1* for single-end and with *--no-novel-juncs -p 4 --bowtie1 -r 30 --mate-std-dev 60* for paired-end to best accommodate observed insert size distribution.

NEUMA-1.2.1 was run with options *--mm = 2 -f = f -d = E -L = 101 -D = 600*, along with Bowtie 1.0.0 with options *--minins 0 --maxins 800 -v 2 -a --suppress 5,6,7 -p 4.*

RSEM 1.2.5 was run with options *-p 4 –time --output-genome-bam --fragment-length-mean 150 --fragment-length-sd 35 --ci-memory 2048 --no-qualities*. Mapping was done using the code provided along with RSEM.

IsoEM 1.1.1 was run after modifying a line in the script to ‘startMem = −Xms10g, maxMem = −Xmx10g for 5 ~ 40M reads and startMem = −xms10g, maxMem = −Xmx100g for over 100M reads’ to avoid using more than existing memory and producing erroneous results. The options were *-m 250 -d 25* after running bowtie 1.0.0 with options *-k 10 -v 2 -f -p 4*. The options were taken from the example file provided by the developers.

For sailfish, indexing was performed using the options -k 20 -o, and quantification was performed using the options -l "T = PE:O= > <:S = SA" -p 4. The default option produces both bias-corrected and uncorrected result.

For performance comparison, all the programs were run on a regular server with 2.90 GHz CPUs on a CentOS 6.3 operating system.

### Real data analysis

Several independent HiSeq2000 RNA-seq data sets were collected on the same sample (UHRR (universal human reference RNA) and HBRR (human brain reference RNA)), for which large-scale qPCR results are available (see Additional file 1: Table S2, S3 for more detail).

For RNA-seq, each program was run with the human transcriptome model of ENSEMBL GRCh37.72 or RefSeq (refgene, downloaded from UCSC on Jul 15, 2014 and cleaned up for duplicate entries). For each gene symbol, all transcripts associated with the gene symbol were summed to obtain the gene-level expression estimates. NEUMA generates gene-level and isoform-level estimate separately. The gene expression level for NEUMA was obtained from gene-level estimates when available, or sum of all isoform levels. We excluded genes whose expression level could not be computed by NEUMA. For IsoEM, option -a auto-fragment-distrib was used instead of -m and -d options for fragment length distribution.

For qRT-PCR, MAQC consortium generated four replicates of TaqMan qRT-PCR on 1001 genes for UHRR and HBRR. Gene-level measurements were provided as 2^Pol2_Ct − gene_Ct^ which could be directly used after log-transformation. We used 841 and 839 genes for which the matching gene symbol exists in the ENSEMBL and RefSeq annotations, respectively. Wang et al. (GSE4214) generated four replicates of TaqMan qRT-PCR on 1363 genes for UHRR. For the Wang et al. data, we directly took the 2^− Ct^ value as the gene-level measurement and did log-transformation. We used the 1287 and 1278 genes with a matching symbol in the ENSEMBL annotation.

The MKN-28 data set was run with the RefSeq annotations. All 27 genes had a matching symbol.

As a measure of accuracy, we used the maximum correlation coefficient between RNA-seq-based gene expression level estimates (computed as log(TPM × *τ* + 1), maximized over τ) and qRT-PCR-based measurements for each method and data set.

## Additional file

Additional file 1: **Supplementary Material.** (PDF 250 kb)

## Abbreviations

RPKM: Reads per kilobase per million reads
FPKM: Fragments per kilobase per million reads
TPM: Transcripts per million
RNA: Ribonucleic acid
EM: Expectation-maximization
CPU: Central processing unit
MB: Megabytes
GB: Gigabytes
GHz: Gigahertz
