# Supplementary material for: EMSAR: estimation of transcript abundance from RNA-seq data by mappability-based segmentation and reclustering
Source: BMC Bioinformatics. 2015 Sep 3;16:278. doi: 10.1186/s12859-015-0704-z (PMC4559005; doi:10.1186/s12859-015-0704-z)
Supplement: Supplementary file 1 — Supplementary Material. (PDF 250 kb) [file 12859_2015_704_MOESM1_ESM.pdf]

Supplementary Material for

EMSAR: Estimation of transcript abundance from RNA-seq data by mappability-based segmentation and reclustering

Soohyun Lee[1,#], Chae Hwa Seo[2,#], Burak Han Alver[1], Sanghyuk Lee[2,3,*], Peter J. Park[1,4*],

[1]Center for Biomedical Informatics, Harvard Medical School, Boston, MA, USA
[2]Emerging Technology Center, DNA link, Seoul, South Korea
[3]Ewha Womans University, Seoul, Korea
[4]Informatics Program, Boston Children's Hospital and Division of Genetics, Brigham and Women's Hospital, Boston, MA, USA

[#] These authors contributed equally.
[*] Corresponding authors.

Supplementary table 1. FLUX Simulator parameters

NB_MOLECULES	50000000
LOAD_CODING	YES
RTRANSCRIPTION	YES
RT_PRIMER	RH
RT_LOSSLESS	YES
RT_MIN	30
RT_MAX	1000
TSS_MEAN	25
POLYA_SCALE	80
POLYA_SHAPE	2
FRAGMENTATION	YES
FRAG_METHOD	UR
FRAG_SUBSTRATE	RNA
READ_LENGTH	101
PAIRED_END	YES
FILTERING	YES
SIZE_DISTRIBUTION	N(300,100)
SIZE_SAMPLING	MH
FASTA	YES

Supplementary table 2. RNA-seq data used for comparison with qRT-PCR

SRA ID	SRA experiment ID	PE/ SE	sample	study	note	total reads	read length (bp)	insert length range (bp)	modal length (bp)
SRR1261168	SRX523771	PE	UHRR	Butterfield [1]		134,921,154	101 x 2	100 ~ 300	125
SRR1261170	SRX523772	PE	UHRR	Butterfield [1]		72,897,482	101 x 2	100 ~ 300	135
SRR515084	SRX155461	PE	UHRR	Wu [2]		28,885,185	91 x 2	180 ~ 220	194
SRR950078	SRX333347	PE	UHRR	Rapaport [3]	1/5 replicates	100,387,010	100 x 2	100 ~ 300	159
SRR950079	SRX333348	PE	HBRR	Rapaport [3]	1/5 replicates	111,037,701	100 x 2	100 ~ 350	175
-	-	PE	MKN-28	Lee [4]		7,573,109	36 x 2	37 ~ 160	104

Supplementary table 3. qRT-PCR data used for comparison with RNA-seq data

	Study	Number of genes	Protocol	Sample	Replicates
UHRR MAQC	MAQC [5]	1001	TaqMan	UHRR	Mean over 4 replicates
UHRR Wang	Wang et al.[6]	1363	TaqMan	UHRR	Mean over 4 replicates
HBRR MAQC	MAQC [5]	1001	TaqMan	HBRR	Mean over 4 replicates
MKN-28 Lee	Lee et all.[4]	27	SYBR	MKN-28	Mean over 3 replicates

Supplementary methods

Modified suffix array that clusters positions of identical sequences on the transcriptome

For computing all the segment lengths in the transcriptome, we modified the concept of a suffix array [29, 30]. EMSAR builds an array of the starting positions of all the substrings of length R on the concatenated transcriptome sequence S. Then, the array is alphabetically sorted by the substrings. For unstranded RNA-seq, S includes both strands of the transcriptome and the array stores the position on S of the alphabetically smaller of a sense substring and its reverse complement (Figure S7a). For strand-specific RNA-seq, the S includes only the sense transcriptome. Linear-time sorting is possible for a suffix array

[31-33], but because of the modification, we used a quick sort algorithm [34], which we also modified for multi-threading.

Data structure for transcriptome index that stores information of all segments

Figure S8a provides a scheme of the structure. The purpose of this structure is to have a hash-like fast access to individual segments given the set of associated transcripts, while using a minimal amount of memory.

An individual segment is implemented as a structure with member variables (effective) segment length, read count and an array of transcript ID's. The transcript ID's are sorted and the first ID is omitted from the structure and instead used as an index to point to a sorted linked list of segment structures that share the first transcript ID. The linked lists are grouped by the number of transcripts in the segments and linked to a pointer array whose index corresponds to the number of transcripts - 2. For segments associated with a single transcript, the transcript array is omitted.

Finding closed sets of transcripts using recursive propagation

Given a set of segments and their associated transcripts, we want to find disjoint sequence-sharing sets of transcripts that are 'connected' by a segment either directly or indirectly. The problem can be solved using a classic connected component algorithm, using a graph representation with transcripts as vertices that are connected when two transcripts share a segment. An alternative graph can be represented by segments as vertices that are connected when two segments share a transcript. For our purpose, both types needed because all the transcripts in a sequence-sharing set is simultaneously estimated using a set of all the segments associated with the transcripts in this set, which equals the equivalence class for segments. In other words, a sequence-sharing set of trasncripts needs to be assigned to a set of transcripts and a set of segments. Therefore, we used an algorithm that does both simultaneously, rather than using a one-way graph structure with either transcript or segment as vertices. The algorithm is reminiscent to the traditional recursive depth-first search [7].

Figure S8b describes the recursive algorithm used for finding a sequence sharing set (sid) and assigning it to a set of transcripts (tid's) and a set of segments (cid's). Two output arrays are generated; CS array, with indices representing cids and elements representing an associated sid, and TS array, with indices representing tids and elements representing an associated sid. For this purpose, it uses two arrays, CT array, with indices representing cids and elements representing a set of tids, and a TC array, with indices representing tids and elements representing a set of cids. Below is a brief description of the algorithm in steps.

> Step 1: start from the first segment (cid=0) and assign the first sequence-sharing set (sid=T=0) to elements of CS array.
>
> Step 2: find all associated transcripts (tid's) from CT array and assign sid=T to elements of TS array.

Step 3: for each of these transcripts, find all associated cid's from TC array.

Step 4: repeat step 1~3 for the cid's found at step 3, until there is no new assignment.

Step 5: Find the next unassigned cid and use a different sid =T+1 and repeat step 1~4.

Step 6: Repeat steps 1~5, until there is no unassigned cid.

Fragment size-weighted segment length for paired-end data and single-end data variable read length.

Given a fixed fragment size d, the expected number of RNA-seq fragments of size d in a segment C is

$$\lambda_{d,c} = \left(\sum_{i \in V(C)} e_i\right) L_{d,c} w_d S \text{ , ...(1)}$$

where e_i is the expression level of transcript i associated with C, $L_{d,c}$ the number of possible positions for fragments of size d in segment C, w_d is the observed probability of fragments of size d and S is the total read count in the RNA-seq data. Since $S' = w_d S$ is the total number of reads of fragment size d in the library, it is equivalent to an imaginary situation in which we're using only a subset of the RNA-seq library with a fixed fragment size and estimating the expected portion of that library that came from a specific segment C.

For convenience of computation, EMSAR merges a range of fragment lengths and uses a single number for λ and L, as follows.

The expected total count of reads in C can be obtained by summing $\lambda_{d,c}$ over all fragment sizes.

$$\lambda_c = \sum_d \lambda_{d,c} \text{ ....(2)}$$

From (1) and (2), we get

$$\lambda_c = \left(\sum_{i \in C} e_i\right)\left(\sum_d L_{d,c} w_d\right) S \text{ ....(3)}$$

Therefore, we define a segment length L_c as below:

$$L_c = \sum_d L_{d,c} w_d \text{ ....(4)}$$

$$\lambda_c = \left(\sum_{i \in C} e_i\right) L_c S \text{ ....(5)}$$

Unambiguous fragment length filtration

EMSAR does not use reads with an ambiguous fragment length, or reads that map to two or more locations on the transcriptome with different fragment lengths. This does not cause underestimation due to missing reads because the positions associated with ambiguous fragment lengths are also excluded from segments. The only way that reads with ambiguous fragment lengths can contribute is by providing information of which fragment length is more likely and how much more likely. A read is more likely to

have come from the transcript to which it is mapped with a fragment length with a higher probability. However, to effectively use this information, an accurate fragment length distribution is critical. An incorrect distribution, (e.g. an assumed Gaussian distribution), could even be detrimental to the accuracy. EMSAR is not sensitive to such deviation of fragment length distribution.

An illustration is shown in Figure S9, as to what types of read are used versus not used (both RNA-seq reads and for identifying segments).

Hill-climbing method

The following is the pseudocode for the hill-climbing algorithm used by EMSAR. In the pseudocode, sid, cid and tid refer to a specific sequence-sharing set, segment and a transcript, respectively. The final estimates are saved in the array FPKM with length equal to the number of transcripts. Function Fp calculates a log likelihood without constant. The global variable CONVEERGENCE_EPSILON_STEPSIZE is the precision that can be specified by the user which is set to 10^{-15} by default.

```
FOR EACH sid

    SET C to the_array_of_cids_associated_with_sid
    SET init_max to 100
    SET init_min to 0
    SET init_stepSize to 100
    SET acc to 2.0

      SET nLoop to 0
      DO
        /* number of iterations, if this is too large, then go back and choose a different initial values */
        SET nIter to 0
        SET notconverged to 0

        /* random initial point betweeen init_min and init_max (FPKM = 'currentPoint') */
        FOR EACH tid associated with sid
           Assign random number to FPKM[tid]
        END FOR

        FOR i = 0 to Number_of_tids_in_sid
        SET stepSize[i] = init_stepSize;

        DO
           INCREMENT nIter
           SET premaxF to Fp(C)
           SET max_stepSize to 0

           FOR i = 0 to Number_of_tids_in_sid

             SET tid to the_ith_tid_in_sid

             IF stepSize[i]<CONVERGENCE_EPSILON_STEPSIZE
                  CONTINUE TO THE NEXT ITERATION OF THE LOOP
             END IF

             SET candidate[0] to 0;
             SET candidate[1] to -stepSize[i]*acc*2
             SET candidate[2] to -stepSize[i]
             SET candidate[3] to stepSize[i]
             SET candidate[4] to stepSize[i]*acc*2

             SET best to -1
             SET maxF to LOWEST_NUMBER

             FOR j = 0 to 4
                SET FPKM[tid] to FPKM[tid]+candidate[j]
                SET F to Fp(C)
                SET FPKM[tid] to FPKM[tid]-candidate[j]
                IF F>maxF
                   SET maxF to F
                   SET best to j
                END IF
             END FOR

             IF best==0
                SET stepSize[i] to stepSize[i]/acc
             ELSE IF best==2 OR best==3
                SET FPKM[tid] to FPKM[tid]+candidate[best]
             ELSE
                SET FPKM[tid] to FPKM[tid]+candidate[best]
                SET stepSize[i] to stepSize[i]*acc
             END IF

             IF stepSize[i] > max_stepSize
                 SET max_stepSize to stepSize[i]
             END IF

           END FOR

           IF nIter> MAX_NITER_MLE
             SET notconverged to 1
             BREAK OUT OF THE LOOP
           END IF

        WHILE Fp(C) - premaxF >= CONVERGENCE_EPSILON OR max_stepSize > CONVERGENCE_EPSILON_STEPSIZE
        INCREMENT nLoop
      WHILE notconverged==1 OR nLoop < MAX_NLOOP_MLE

END FOR
```

1. Butterfield YS, Kreitzman M, Thiessen N, Corbett RD, Li Y, Pang J, Ma YP, Jones SJ, Birol I: **JAGuaR: junction alignments to genome for RNA-seq reads.** *PLoS One* 2014, **9:**e102398.
2. Wu J, Zhang W, Huang S, He Z, Cheng Y, Wang J, Lam TW, Peng Z, Yiu SM: **SOAPfusion: a robust and effective computational fusion discovery tool for RNA-seq reads.** *Bioinformatics* 2013, **29:**2971-2978.
3. Rapaport F, Khanin R, Liang Y, Pirun M, Krek A, Zumbo P, Mason CE, Socci ND, Betel D: **Comprehensive evaluation of differential gene expression analysis methods for RNA-seq data.** *Genome Biol* 2013, **14:**R95.
4. Lee S, Seo CH, Lim B, Yang JO, Oh J, Kim M, Lee B, Kang C: **Accurate quantification of transcriptome from RNA-Seq data by effective length normalization.** *Nucleic Acids Res* 2011, **39:**e9.
5. Shi L, Reid LH, Jones WD, Shippy R, Warrington JA, Baker SC, Collins PJ, de Longueville F, Kawasaki ES, Lee KY, et al: **The MicroArray Quality Control (MAQC) project shows inter- and intraplatform reproducibility of gene expression measurements.** *Nat Biotechnol* 2006, **24:**1151-1161.
6. Wang Y, Barbacioru C, Hyland F, Xiao W, Hunkapiller KL, Blake J, Chan F, Gonzalez C, Zhang L, Samaha RR: **Large scale real-time PCR validation on gene expression measurements from two commercial long-oligonucleotide microarrays.** *BMC Genomics* 2006, **7:**59.
7. Michael T. Goodrich RT: *Data Structures and Algorithms in Java.* 5th edn: Wiley; 2010.

Supplementary Figures

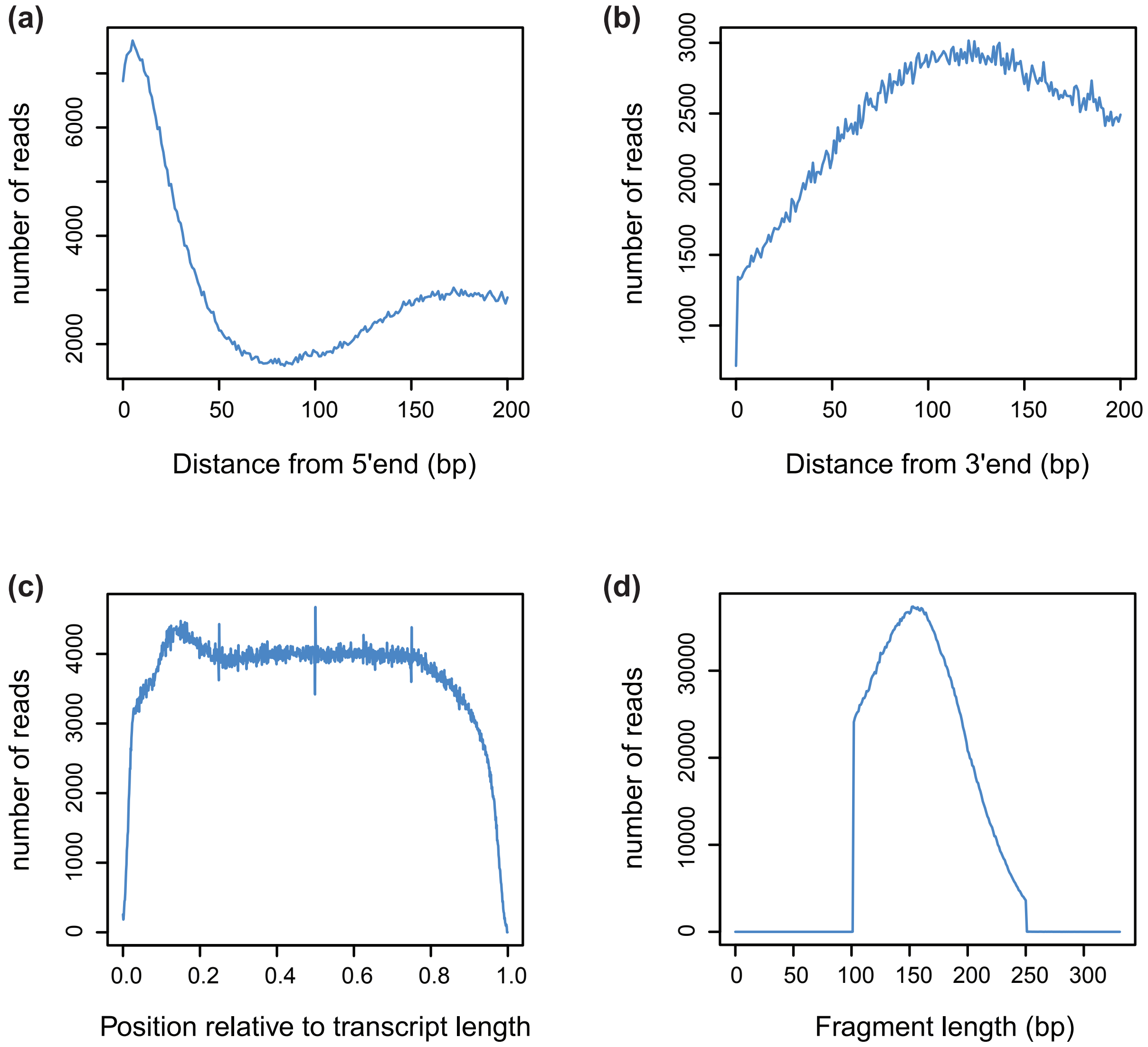

Figure S1. Distribution of read positions and fragment length in the simulated data. (a) Distance from 5'end, **(b)** distance from 3'end, **(c)** relative position within a transcript, **(d)** fragment length, from one of the simulated data sets with 5 million reads each. The plots were obtained directly from the information from the simulated reads not from the mapping, and thus do not include ambiguity introduced by alignment.

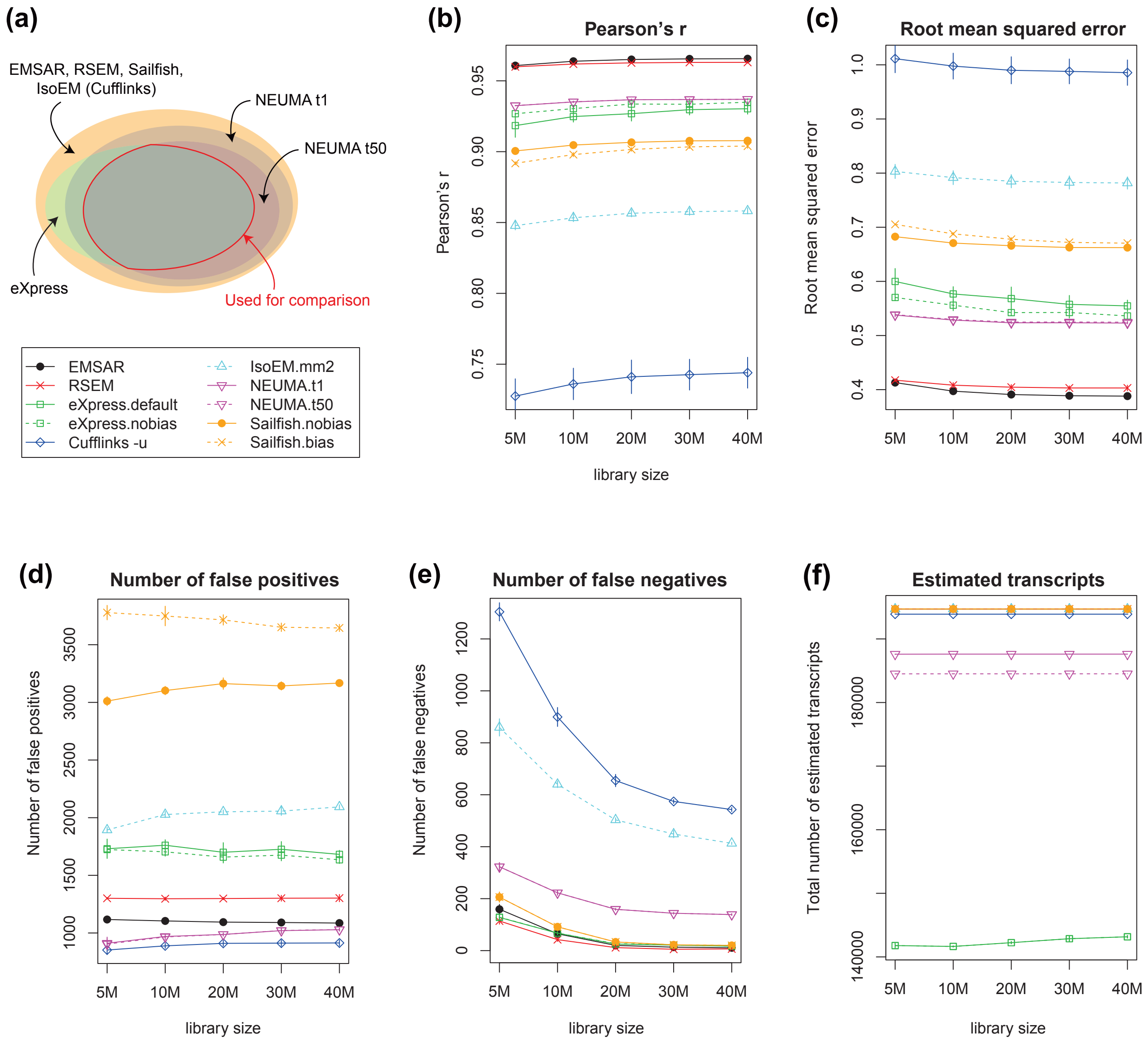

Figure S2. Comparison of accuracy across multiple methods for paired-end RNA-seq. (a) A schematic diagram showing the number of isoforms estimated by individual methods and the commonly estimated ones. Diagrams are not drawn in exact scale. **(b)-(e)** Four different evaluation criteria are applied to multiple quantifiers on the common set of isoforms indicated in (a). **(b)** Pearson correlation coefficient **(c)** root mean squared error **(d)** number of false positives, i.e., isoforms with zero true expression and with 1 or larger value in log(estimated_TPM· σ+1), **(e)** number of false negatives, i.e. isoforms with 1 or larger value in log(true_TPM+1) and zero estimated expression. **(f)** total number of transcripts whose expression level is reported. EMSAR, RSEM, Sailfish and IsoEM report all transcript levels. Error bars indicate standard deviation, not standard error of mean.

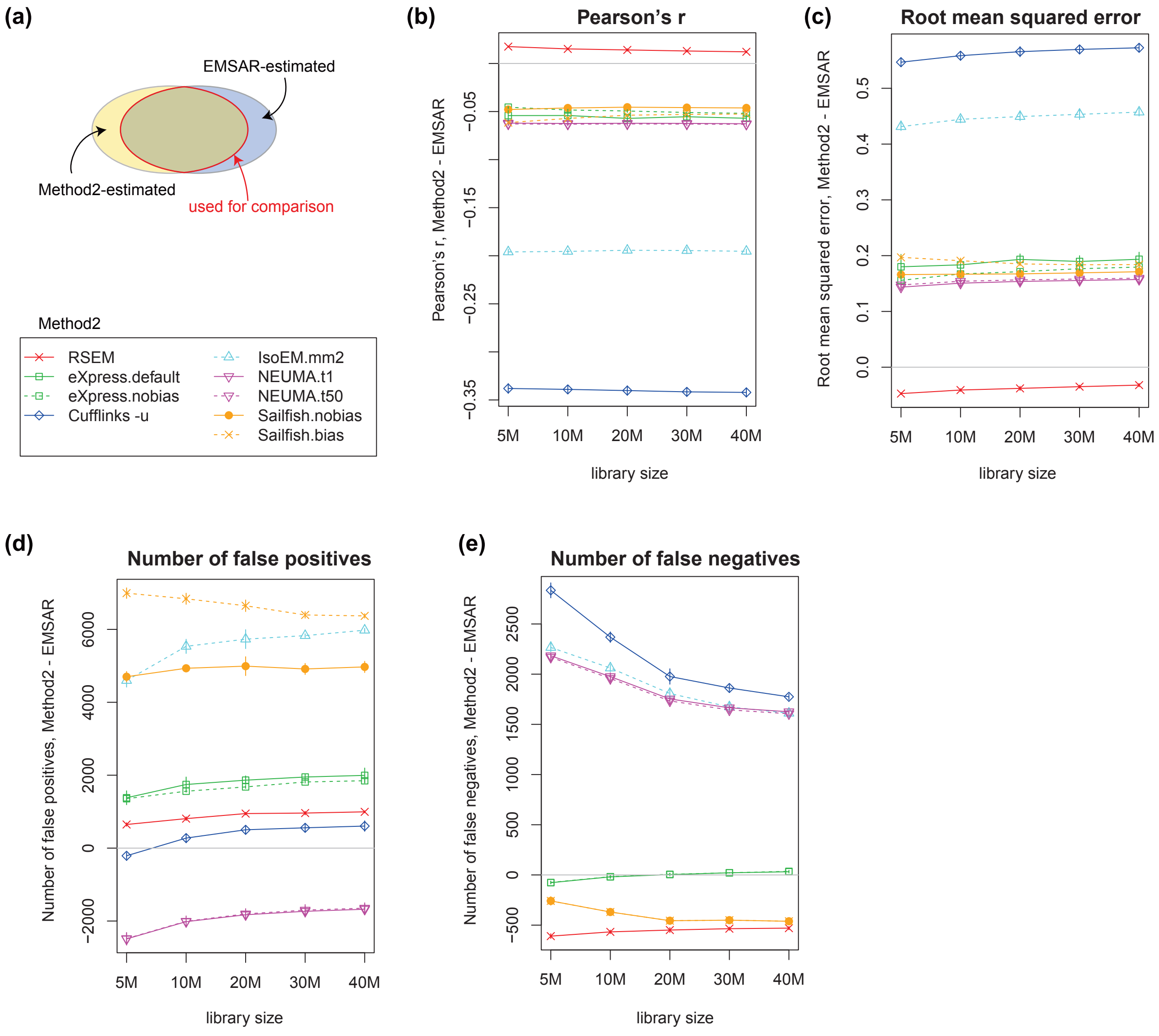

Figure S3. Pairwise comparison of accuracy between EMSAR and another method for paired-end RNA-seq. (a) A schematic diagram showing the number of isoforms estimated by individual methods and the commonly estimated ones. Diagrams are not drawn in exact scale. **(b)-(e)** Figure scheme is the same as in Figure S2 except that the differences of the method of choice and EMSAR are plotted.

(a) Single-end

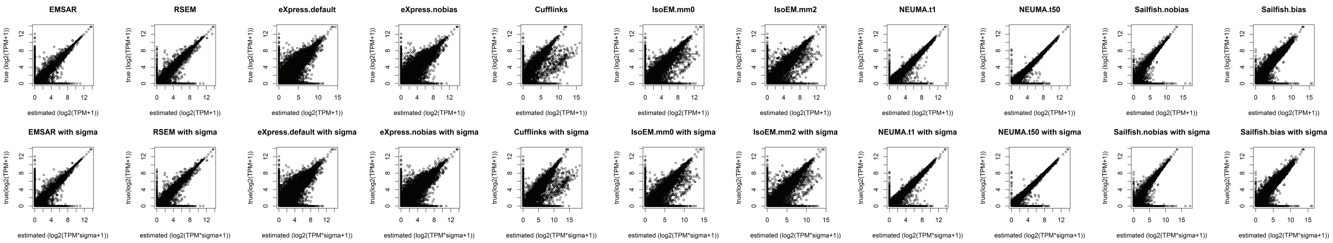

(b) Paired-end

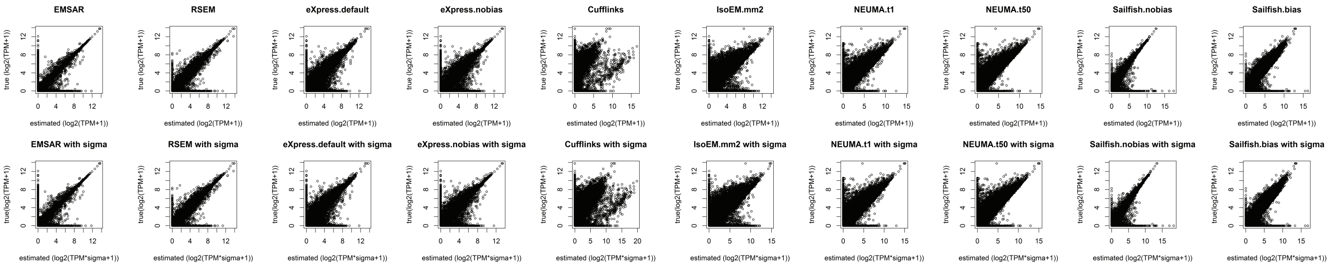

Figure S4. Scatter plots showing true and estimated expression levels from various programs. log(true_TPM+1) versus either log(estimated_TPM+1) (upper panel) or log(estimated_TPM$\cdot$ σ+1) (lower panel) for one of **(a)** single-end and **(b)** paired-end simulation data with 40 million reads each.

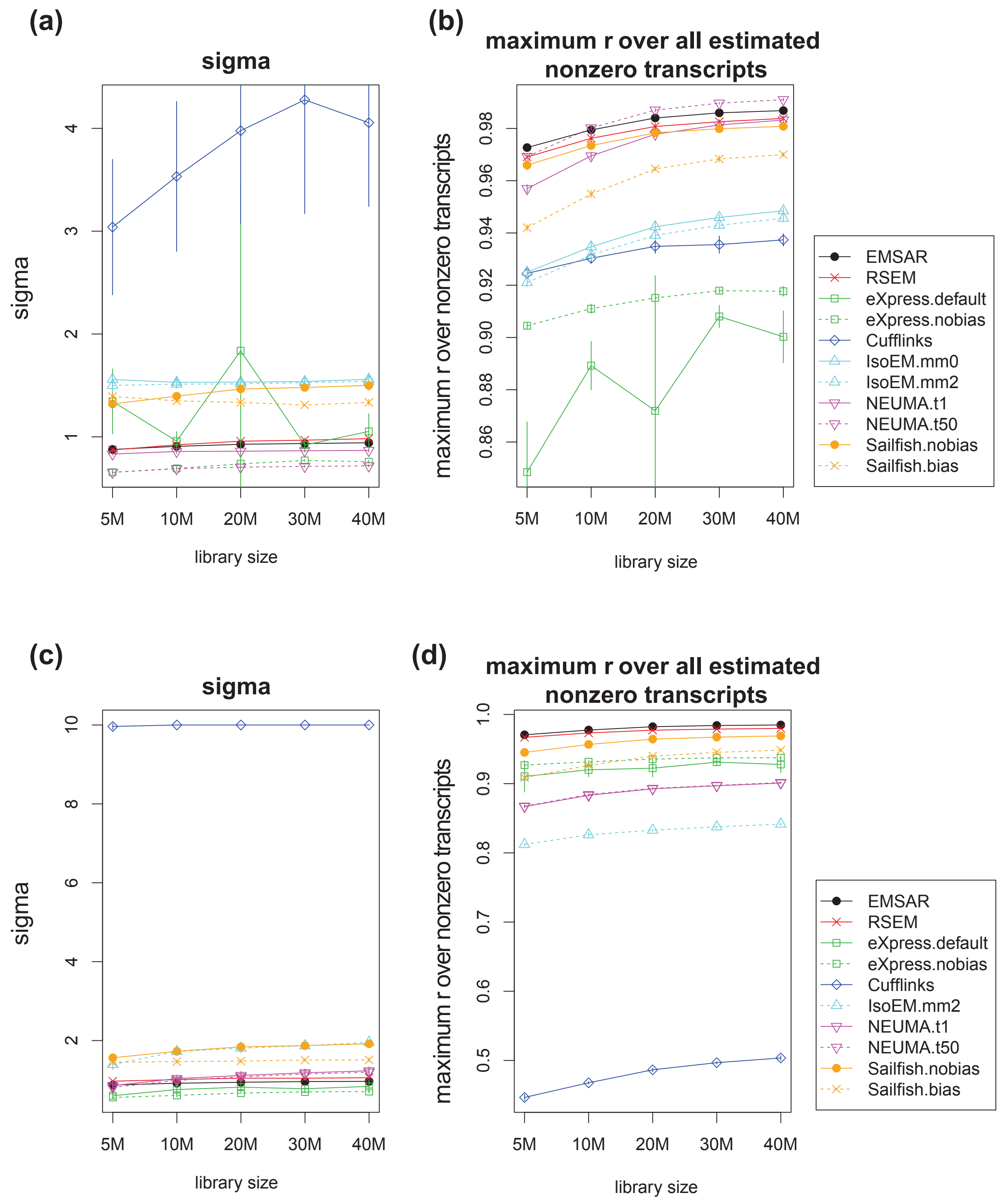

Figure S5. Scale factor and maximum correlation coefficient in nonzero transcripts. (a,b) Single-end simulated RNA-seq data, **(c,d)** paire-end simulated RNA-seq data. **(a,c)** Chosen scale factor σ that maximizes correlation coefficient among all nonzero estimated and nonzero expressed transcripts for each method on each data set. **(b,d)** the maximum correlation coefficient obtained by using σ. Note that for the actual comparisons, zero-estimated and zero-expressed transcripts are included.

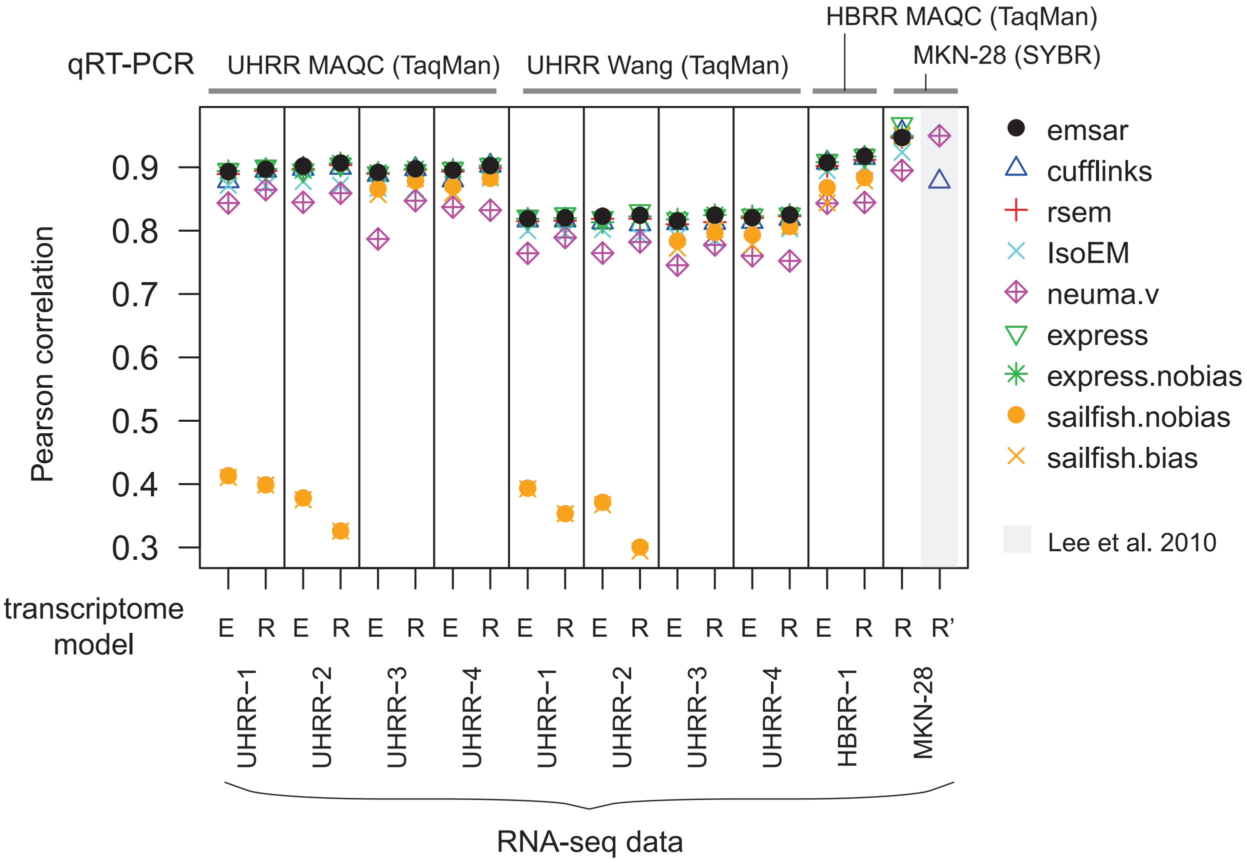

Figure S6. Comparison of accuracy as measured by concordance to qRT-PCR on real RNA-seq data. The same figure 6, except the inclusion of Sailfish. Pearson correlation between RNA-seq-based gene expression level estimates (log(TPM*τ+1)) and qRT-PCR-based measurements (ΔCt), across independently performed experiments. τ was used to maximize the correlation to adjust for any scale effect of the pseudocount, which allowed inclusion of zero estimates. Six RNA-seq data sets (UHRR-1 to -4, HBRR-1, MKN-28) and four qRT-PCR sets (UHRR MAQC [19], HBRR MAQC [19], UHRR Wang et al. [20], MKN-28 from Lee et al. [6]) were compared. RNA-seq-based quantification was run on two independent transcriptome models (ENSEMBL (E) and RefSeq (R)). The results from Lee et al. on an older version of RefSeq model (R') was shown to the right for comparison (grey box). Gene-level estimates were obtained by summing the relevant isoform-level estimates, except for NEUMA, for which gene-level estimates were obtained either from the reads common to all the isoforms of a gene or by summing the isoform-level estimates derived from reads unique to individual isoforms.

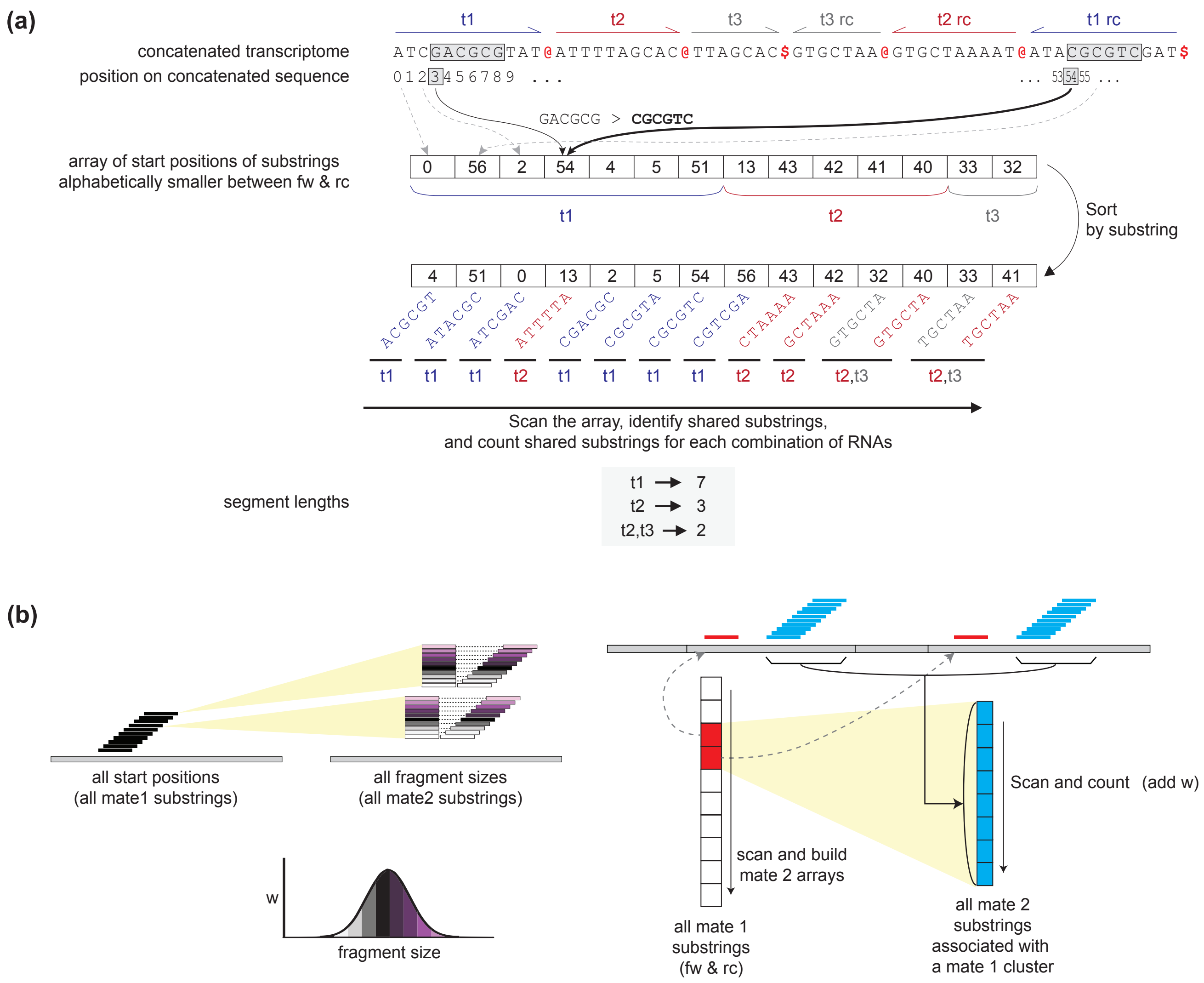

Figure S7. Detailed algorithm description of EMSAR. (a) The algorithm of computing segment lengths for unstranded single-end RNA-seq is illustrated using a simplified transcriptome. First, all the transcript sequences (in this case, three) are concatenated followed by their reverse complementary sequences. Then, an array is created to store the start positions of substrings of a specific length (in this case, 6 bp) on the concatenated sequence. The length of the substrings is identical to the read length. Any substring that contains a delimiter character (e.g. '@' or '$') is skipped. Only the alphabetically smaller between a sense substring and its revere complement is stored (eg. position 54 is stored but not 3.). This is to ensure that a substring and its reverse complement are treated as indistinguishable, like the reads from an unstranded RNA-seq experiment. Then, this array is sorted by the associated substrings, to identify clusters of identical substrings and count the shared substrings for each unique combination of transcripts. **(b)** A simplified algorithm of computing segment lengths for paired-end RNA-seq. First an array containing start positions of all substrings of a specified length is created and sorted, which serves as a mate1 array. Then, for every cluster of identical mate1 substrings, a mate2 array of all start positions that can be paired with mate1 is created within a specified fragment length range, and is sorted by the mate2 substrings. For unstranded data, the mate2 array only includes cases in which the second mate is alphabetically smaller than its reverse complement. The mate2 array is removed after being scanned for counting shared substrings. The length of segment is computed for individual fragment lengths during indexing, and later weighted by the data-specific fragment size distribution.

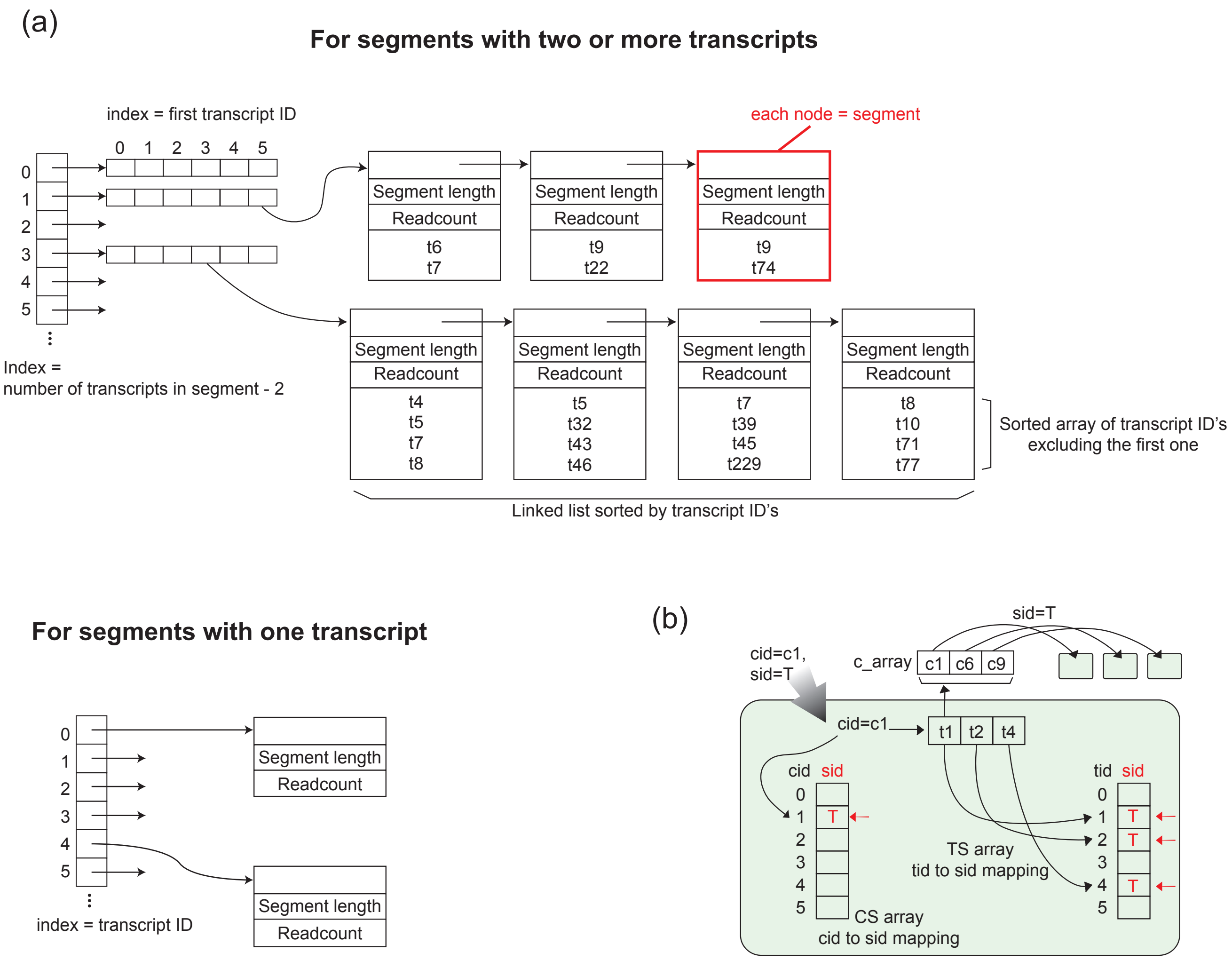

Figure S8. Algorithmic details of EMSAR. (a) The data structure for transcriptome index. The data structure is constructed as the suffix array is scanned, can be stored as an index file and reconstructed from the index file, and is used to count reads for each segment as the program reads through RNA-seq alignments. The structure uses relatively stable amount of memory and allows relatively fast access to individual segments. For segments associated with multiple transcripts, the number of transcripts (tsize) and the first transcript ID (tid0) are directly accessed as indices of a two-dimensional array that stores the pointer to a linked list of all segments with the corresponding tsize and tid0. An individual node in the linked list corresponds to a segment and it contains the rest of the transcript ID's (tid's) associated with the segment, as a numerically sorted array. Each node also stores a read count and an array of segment lengths computed for individual fragment sizes. The linked list is sorted by the tid array, as it is created. For segments that consist of a single transcript, a one-dimensional array is used with indices representing transcript IDs. **(b)** A recursive propagation strategy used for identifying sets of segments. cid: segment ID; tid: transcript ID; sid: sequence-sharing set ID. The first segment (cid=0) is fed to the recursion, in which cid is assigned sid=T and all of its associated tids are also assigned sid=T. Then, for each of the tid's, all of its associated cid's is fed to the same function with sid=T. This process is repeated until no new assignment is possible. Then, pick the next unassigned cid and do the same with sid=T+1. Do this until no more unassigned segment is available.

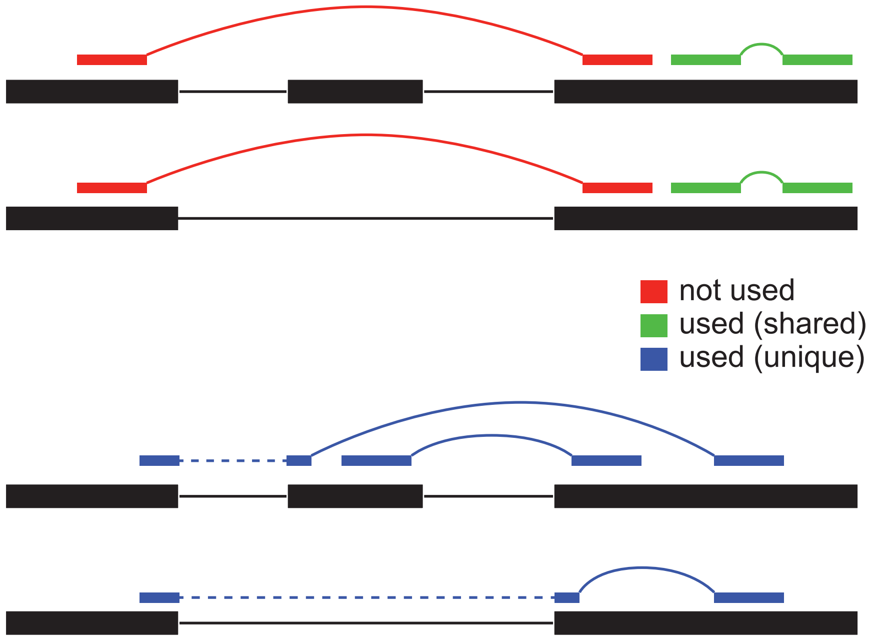

Figure S9. Reads with ambiguous fragment size. An example illustration of two isoforms, one with and one without a skipped exon. Top: Two different types of shared reads are shown. The one with unambiguous fragment size (green) is used by EMSAR, whereas the one with ambiguous fragment size (red) is not. Bottom: Various types of transcript-specific reads that are always used by EMSAR (blue).
